# Children and Adolescents with Co-Occurring Attention-Deficit/Hyperactivity Disorder and Autism Spectrum Disorder: A Systematic Review of Multimodal Interventions

**DOI:** 10.3390/jcm14114000

**Published:** 2025-06-05

**Authors:** Carmela De Domenico, Angelo Alito, Giulia Leonardi, Erica Pironti, Marcella Di Cara, Adriana Piccolo, Carmela Settimo, Angelo Quartarone, Antonella Gagliano, Francesca Cucinotta

**Affiliations:** 1 I.R.C.C.S. Centro Neurolesi Bonino Pulejo, 98124 Messina, Italy; carmela.dedomenico@irccsme.it (C.D.D.); marcella.dicara@irccsme.it (M.D.C.); adriana.piccolo@irccsme.it (A.P.); carmela.settimo@irccsme.it (C.S.); angelo.quartarone@irccsme.it (A.Q.); 2 Department of Biomedical, Dental Sciences and Morphological and Functional Images, University Hospital “G. Martino”, 98124 Messina, Italy; alitoa@unime.it; 3 Department of Physical and Rehabilitation Medicine, University Hospital “G. Martino”, 98124 Messina, Italy; giulia.leonardi@polime.it; 4 Woman-Child Department, Unit of Child Neurology and Psychiatry, Policlinico Riuniti Foggia, 71122 Foggia, Italy; erica.pironti@gmail.com; 5 Child and Adolescent Neuropsychiatry, Department of Medicine and Surgery, “Kore” University of Enna, 94100 Enna, Italy

**Keywords:** Autism spectrum disorder, attention deficit disorder with hyperactivity, treatment, intervention, psychopharmacology, drug therapy, behavior therapy

## Abstract

**Background/Objectives**: The co-occurrence of Attention-deficit/hyperactivity Disorder (ADHD) and Autism Spectrum Disorder (ASD) is very common and worsens adaptive functioning. This systematic review evaluates both pharmacological and non-pharmacological interventions in this underserved population. **Methods**: Registered on PROSPERO (CRD42024526157), a systematic search was conducted on PubMed, Embase, and Web of Science until 5 April 2025. The review includes (a) pilot studies and RCTs, (b) participants aged <18 years, (c) diagnoses of ASD and ADHD based on DSM-IV/V or ICD-9/10, (d) at least one group receiving any intervention, and (e) publications in English, Italian, Spanish, or German. Newcastle Ottawa Scale tools for non-randomized studies and the Cochrane Risk of Bias Tools for randomized controlled trials were used to assess studies’ quality. **Results**: A total of 32 studies were included: 87.5% concerning pharmacological treatments. Specifically, methylphenidate (MPH, n = 11), atomoxetine (ATX, n = 11), guanfacina (n = 4), clonidine (n = 1), or atypical antipsychotics (n = 1) were examined. MPH and ATX were most frequently studied, with both showing positive effects in reducing ADHD core symptoms compared to placebo. ATX also reduces stereotyped behaviors and social withdrawal, although more withdrawals due to adverse events (AEs) were reported for ATX than MPH. Four studies (12.5%) examined non-pharmacological interventions, including treatment with virtual reality tools, digital platforms, educational animations, and biomedical protocols; improvements in emotion recognition, behavioral regulation, attention, and social functioning were found. **Conclusions**: While limited data prevent definitive conclusions, MPH and ATX appear to be relatively safe and effective on hyperactivity-impulsivity symptoms, even in individuals with ASD. Evidence on non-pharmacological treatments is limited, and further studies are needed to better establish their therapeutic potential.

## 1. Introduction

Autism spectrum disorder (ASD) is a neurodevelopmental disorder characterized by impairments in social interaction, communication, restricted and repetitive patterns of behavior, and interests [1].

Its prevalence has risen steadily: recent U.S. surveillance data estimate rates of 1.70% in four-year-olds and 1.85% in eight-year-olds—approximately 1 in 36 children, according to DSM-5-TR criteria.

Across Europe, reported prevalence ranges from 0.38% to 1.55% [2]. Co-occurring psychiatric or mental health conditions are common: about 70% of autistic individuals receive at least one additional psychiatric disorder [3,4].

The high rate of comorbidity may reflect overlapping symptomatology [5], the presence of distinct psychiatric disorders [6], and early life experiences related to the autism spectrum [7]. Among these co-occurring conditions, attention-deficit/hyperactivity disorder (ADHD) is one of the most frequently observed, affecting 30–40% of autistic children [8], and evidence suggests a notable increase in the co-occurrence of these two conditions in comparison with the past [9]. While ASD and ADHD have distinct diagnostic criteria and are classified as separate disorders according to the Diagnostic and Statistical Manual of Mental Disorders (DSM) [1], they share several characteristics, including neuropsychological traits and some genetic influences [10,11,12,13].

Specifically, ADHD is one of the most prevalent neurodevelopmental disorders globally, affecting an estimated 5% to 7.1% of children and adolescents and around 2.5% of adults [14,15,16]. This condition places a significant care burden on both society and families [17,18]. An international consensus statement from the World ADHD Federation summarizing 208 evidence-based findings on ADHD, which highlighted the high prevalence of co-occurring psychiatric disorders, including ASD, and the need for personalized treatment approaches [19].

However, the clinical management of individuals with both ASD and ADHD presents significant challenges. Although methylphenidate (MPH) is widely recommended as a first-line treatment for children and adolescents with ADHD [20], its clinical benefit in patients with co-occurring ASD appears to be reduced, with response rates of 50–60% compared to 75% in individuals with ADHD alone [21,22]. Improvements tend to be greater for hyperactivity–impulsivity than for inattention, and adverse events are reported more frequently in the comorbid population [21]. Overall, treatment response to psychotropic medications in this group is remarkably heterogeneous, and sensitivity to side effects is often high [23].

Because co-occurrence of ASD and ADHD is associated with poorer adaptive functioning and lower quality of life than either disorder alone [24], there is an urgent need to identify effective and targeted interventions [25]. Despite growing interest, evidence for both pharmacological and non-pharmacological options remains limited and fragmented.

For example, recent comprehensive reviews highlight inconsistent findings regarding the safety of ADHD medications in special populations [20] and the limited effectiveness of social skills training in ADHD individuals [26]. These gaps underscore the need for a systematic review that integrates both pharmacological and non-pharmacological approaches. This review, through an integrative approach, aims to provide a comprehensive overview of current therapeutic options for co-occurring ASD and ADHD, helping clinicians and researchers understand existing evidence-based data.

This systematic review aims to address current gaps in the literature by providing a comprehensive synthesis of both pharmacological and non-pharmacological interventions for individuals with co-occurring ADHD and ASD. To the best of our knowledge, this is the first systematic review to simultaneously summarize the existing evidence on both treatment modalities in this often under-recognized population.

Specifically, we aimed to (a) describe the characteristics of the study populations included; (b) identify the symptom domains assessed and the outcome measures employed; (c) evaluate the reported outcomes and safety of pharmacological and non-pharmacological interventions; and (d) examine potential moderators of treatment response, including symptom-specific intervention strategies.

## 2. Materials and Methods

### 2.1. Data Source and Search Strategy

The protocol of this review was registered in the PROSPERO database with the following code: CRD42024526157, in accordance with the Preferred Reporting Items for Systematic Reviews and Meta-Analyses (PRISMA) guidelines [27,28] (see Table S1 in Supplementary Materials for PRISMA Statement). We conducted a systematic search on three electronic databases: PubMed, Embase, and Web of Science. A systematic search was carried out through the combination of the words: “Autism”, “ADHD”, and “Treatment” with the use of various synonyms in the search query or the respective MeSH term; the complete search strategy is reported in Supplementary Table S1. We also searched the reference lists of included studies or reviews to ensure that a comprehensive list of relevant articles was considered for inclusion.

The search strategy included studies focused on co-occurring ASD and ADHD in subjects aged between 2 and 18 years until April 5, 2025. We included studies that met all of the following inclusion criteria: (a) pilot studies, randomized controlled studies (RCTs); (b) studies involving participants aged <18 years; (c) inclusion of individuals with both ASD and ADHD diagnoses according to the DSM IV or 5 [1,29] or the International Classification of Diseases 9 or 10 [30,31] (d) at least a group receiving a pharmacological/non-pharmacological intervention; (e) English, Italian, Spanish, and German languages. The exclusion criteria were: (a) clinical cases, preprints, letters to the editor, reviews, and systematic reviews; (b) studies with a sample that did not have co-occurring diagnoses of ASD and ADHD; and (c) studies in languages other than English, Italian, Spanish, and German.

### 2.2. Selection Procedures

Study selection was conducted by two blinded authors (CDD, MDC). After removing duplicates, identified references were initially screened by title and abstract. Full-text articles were then assessed for eligibility based on title, abstract, full-text content, and specificity of the topic. In cases of disagreement, a third author (FC) was consulted to reach a consensus. When overlapping studies were identified, the largest study was included.

### 2.3. Data Extraction and Evaluation

All variables were coded as categorical and well-defined by one author (FC) to accurately describe and assess study characteristics. The coding process included interim and final agreement checks to ensure consistency and reliability. After initial coding, two blinded authors (CDD, MDC) independently reviewed and aligned their assessments with the predefined metrics. Any discrepancies identified during the interim checks were discussed and resolved by consensus between the authors, ensuring alignment before proceeding. In the final step, a thorough review confirmed full agreement on the coding and summary of the articles. The data extraction process included background information such as first author, publication year, country, and study design. Sample characteristics were also analyzed, including sample size, ethnicity, gender distribution (M:F ratio), age (mean, standard deviation, and range), clinical diagnostic assessments, comorbid neuropsychiatric conditions, reported Intellectual Quotient (IQ), and the size of each experimental group, where applicable. Intervention characteristics were examined regarding objectives and main effects, type of intervention, dosage (mean or range), duration, adverse events, and outcome measures.

### 2.4. Strategy for Data Synthesis

Primary outcomes included intervention effects and improvement in core symptoms (e.g., inattention, hyperactivity, and social behavior). Secondary outcomes included safety (adverse events), tolerability (e.g., dropout rates), and treatment response moderators. A meta-analysis was not conducted due to substantial clinical and methodological heterogeneity among the included studies, including differences in sample characteristics, intervention types, outcome measures, and study designs.

Additional sub-analyses were conducted based on: (1) sample characteristics, (2) domains investigated and outcome measures type of intervention, and (3) safety and tolerability, considered as the proportion of participants who left the study because of any side effect. (4) synthesis of main effects on studied intervention, (5) response moderators, and (6) quality assessment.

No statistical analysis of heterogeneity was performed due to the high variability in study designs, interventions, and outcome measures.

### 2.5. Quality Assessment

The Newcastle-Ottawa Scale (NOS) [32] was used to evaluate the quality of non-randomized studies. The NOS examines eight domains of bias, with each domain receiving a maximum of one star, except for comparability, which can achieve up to two stars. These domains are (1) representativeness of the exposed cohort; (2) ascertainment of exposure; (3) selection of the unexposed cohort; (4) demonstration that the outcome of interest was not present at baseline; (5) comparability of cohorts based on design or analysis; (6) outcome assessment; (7) sufficient follow-up duration; and (8) adequacy of follow-up. Scores for studies range from 0 to 9 points and are divided into the following categories: 0–3, indicating poor quality; 4–6, fair quality; and 7–9, good or high quality.

For randomized controlled trials (RCTs), we used the Cochrane Risk of Bias tool [33], which evaluates seven key domains such as the randomization process; allocation concealment; blinding of participants and personnel; blinding of outcome assessment; incomplete outcome data; selective reporting; and other biases. The results were then standardized according to Agency for Healthcare Research and Quality (AHRQ) criteria [34], classifying RCTs as ‘good quality’, ‘fair quality’, or ‘poor quality’.

Two independent reviewers (GL and AA) conducted the risk of bias assessments. Any discrepancies were discussed with a third reviewer (FC) to reach a consensus. The domains evaluated for each study are detailed in the results table.

## 3. Results

**Figure 1:**
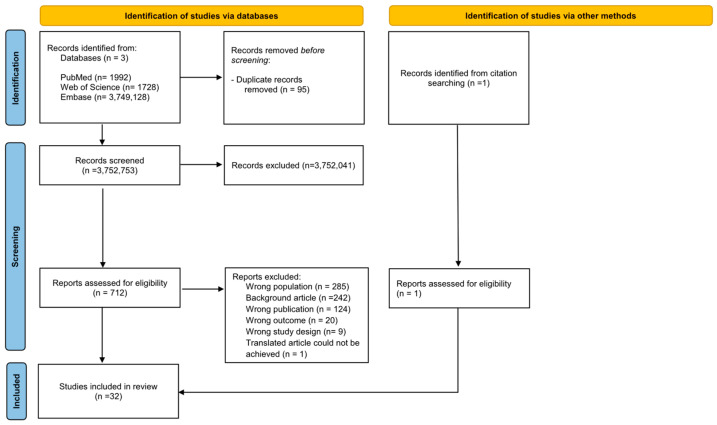
PRISMA 2020 flow diagram.

The literature search yielded a total of n = 3,752,848 results. All the selection processes are reported in Figure 1. After removing n = 95 duplicates, we excluded n = 3,752,041 following the screening procedure; we identified n = 712, which were assessed for eligibility. Finally, we included n = 32 studies, among those n = 28 (87.5%) regarding pharmacological treatment and n = 4 (12.5%) regarding other types of intervention. Most studies (n = 18, 56.25%) were carried out in North America, followed by Europe (n = 10, 31.25%) and Asia (n = 4, 12.5%). A full reference list for the studies included is reported in the Supplementary Materials.

### 3.1. Sample Characteristics

The overall database, considering all studies, comprised n = 1080 subjects with both ASD and ADHD. The sample size of each study ranged from 8 to 128, and the age range of the study participants was 4 to 18 years (mean age 9.5 ± 1.7 years). Of the participants, 63.33% were White, with male gender prevalence. A total of 12 studies (37.5%) included a sample with IQ > 70, and only four (12.5%) studies included IQ < 70, while the other studies (n = 14, 43.75%) included a mixed sample; only n = 2 studies (6.25%) did not refer to the IQ of the participants. All sample characteristics of the included studies are presented in Table 1.

### 3.2. Domains Investigated and Outcome Measures

All studies evaluated ADHD core symptoms as the primary aims of treatment, considering change in severity of attention, hyperactivity, and impulsivity as the main measures of outcome. Specifically, hyperactivity and inattention symptoms were the prevalent therapeutic effects in almost all the studies, at 31/32 (96.9%) and n = 30/32 (93.7%), respectively. Among ASD symptoms, the most investigated domains concerned stereotyped behaviors (n = 24, 75%) and social withdrawal (n = 22, 68.7%). Improvements in stereotyped behaviors were observed only in nine studies out of 24 (37.5%), and social withdrawal in 7/22 (31.8%).

Overall, very heterogeneous outcome measures were used, with a greater prevalence of questionnaires; the most used were: Aberrant Behavior Checklist (ABC; n = 15/32, 46.9%) [62], Conners Parent Rating Scales–Revised (CPRS; n = 10/32, 31.2%) [63], Swanson, Noland, and Pelham Scale IV (SNAPIV; 7/32, 21.9%) [64]. Moreover, clinical observation was performed in most of the studies, with Clinical Global Improvement as the most frequently measured (CGI; n = 19/32, 59.4%) [65]. A complementary view to clinicians’ ratings was collected through teachers’ ratings. In 40.6% (13/32) of the studies, teacher-reported outcome measures were included to assess treatment effects in the school setting. For more details, see Table 2.

For the results’ greater readability, we reported the results of pharmacological and non-pharmacological interventions separately.

### 3.3. Safety

AEs related to pharmacological interventions were investigated using non-standardized parent-reported surveys in 15 out of 28 studies (53.6%), while validated questionnaires were used in only 4 studies (14.3%). Basic physical examinations were conducted in 13 studies (46.4%), most frequently including blood pressure (11 studies, 39.3%), heart rate (10, 35.7%), body weight (10, 35.7%), and electrocardiograms (5, 17.9%). Laboratory testing was conducted in only 2 studies (7.1%), and 4 studies did not report any safety assessment procedures. Regarding overall tolerability, 85 out of 1080 participants (7.9%) discontinued treatment due to adverse events.

A summary of the main adverse events and treatment discontinuation rates by pharmacological agent is provided below:

- MPH: Most frequent AEs were decreased appetite, sleep disturbances, irritability, and gastrointestinal symptoms. 28 out of 388 patients (7.2%) discontinued.
- ATX: Similar profile to MPH, with 45 dropouts out of 306 patients (14.7%).
- GFC: Common AEs included irritability and sleep problems (notably insomnia and mid-sleep awakenings). 2 out of 87 patients (2.3%) withdrew.
- Clonidine: Drowsiness and irritability were reported; no dropouts.

Antipsychotics: Increased appetite and weight gain were observed; no treatment discontinuation. More detailed information on safety and treatment withdrawal is reported in Table 3.

The most reported side effects of MPH were decreased appetite and sleep alterations. Although less frequently, gastrointestinal symptoms and irritability have also been reported. The total withdrawal for side effects was n = 28 (7.2% of a total MPH-treated sample of n = 388 patients). Decreased appetite, sleep difficulties, and gastrointestinal symptoms were also found to be the most frequent side effects in ATX-treated patients. Side effects led to the withdrawal of 45/306 patients treated (14.7%).

The AEs described in GFC trials were irritability in terms of anger outbursts and low frustration tolerance. Moreover, sleep disorders were stated, particularly insomnia, and mid-sleep awakenings were mostly reported. Despite these reported side effects, only 2 patients withdrew due to AEs out of 87 (2.3%).

The clonidine study also reported difficulty sleeping, specifically drowsiness and irritability. The study on antipsychotic treatment reported increased appetite and weight gain as the main concerns. No withdrawals were reported. The safety measures and AEs of all studies are shown in Table 3.

### 3.4. Non-Pharmacological Intervention

In our systematic review, we identified only four studies that focused on non-pharmacological treatments for children and adolescents with co-occurring ASD and ADHD. These studies explored different types of interventions: the first study explored the effects of 15 animated episodes in enhancing emotion recognition; the second study used an immersive interactive video-game technology intervention on preschoolers; the third evaluated a multidimensional treatment protocol; and the last used a digital cognitive training program.

The first RCT study, conducted by Chan et al. [66], used the Transporters intervention, which consists of 15 animated episodes that patients watch daily for a month. Through stories featuring vehicles and characters with human faces expressing emotions, the preschooler patients would learn to recognize emotions in social interactions. Efficacy was assessed through the Emotion Vocabulary Task and Emotion Recognition Tasks—levels of generalization. Authors reported significant improvement in emotion recognition in both ASD and ASD + ADHD groups vs. control, with a good degree of generalization to novel contexts.

The use of virtual reality-based cognitive-behavioral therapy (VR-CBT) with interactive games projected on the floor to train attention, inhibition, and social skills compared with a structured therapist-led program (LSP) that adapts learning strategies to individual profiles was evaluated in an RCT study by Chu et al. [67]. A total of 27 ADHD + ASD preschooler children underwent a total of 40 sessions (2×/week; 20 min VR-CBT + 1 h LSP per session for 20 weeks), while 26 ADHD + ASD preschooler children completed 40 sessions (two sessions per week; 1 h of LSP per session) in 20 weeks. Through ABC, CARS, ADHD-RS-IV, and Go/No-Go tasks, the author showed a significant reduction in hyperactivity-impulsivity symptoms and total scores. No significant change in inattention symptoms was reported.

Patel et al. [68], in an open-label observational study, examined the effects of a multidimensional intervention implemented over three to six months in a small cohort of ten children aged 4 to 10 years. In addition to standard behavioral and educational therapies, this protocol included a range of interventions to attenuate symptoms through an integrative approach. The intervention included environmental control strategies designed to minimize exposure to potential triggers, the introduction of a biological diet, gastrointestinal support therapies, and antigen injection therapy. It also included nutritional supplementation, chelation therapy, and administration of glutathione and methylcobalamin to support mitochondrial function and neurochemical balance. The effects of this intervention were assessed via a personalized questionnaire completed by parents, teachers, and physicians. They reported a general improvement in social interaction, attention, writing, language, and behavioral regulation. Additionally, laboratory analyses demonstrated a significant reduction in urinary lead levels, suggesting a potential benefit of chelation therapy in reducing heavy metal load. Despite these promising results, the lack of a control group and the small sample size limit the generalizability of the results, highlighting the need for further studies to validate these hypotheses.

The second study, conducted by Yerys et al. [69], was a randomized controlled trial investigating the effectiveness of a cognitive training program based on a multi-tasking video game known as “Project Evo”. This intervention consisted of a series of cognitive, perceptual discrimination, attention, and memory tasks, along with continuous visuo-motor coordination exercises administered via a tablet-based platform. The study included a sample of nineteen children, predominantly male, between the ages of 9 and 13. Participants were randomly assigned to either the experimental or control group, with the intervention group completing twenty traditional training sessions of a multi-tasking digital training session over four weeks. Compared to the control group, children who underwent experimental training showed significant improvements in multiple cognitive and behavioral domains, as reflected in the ADHD-RS index scores. Specifically, improved behavioral regulation, emotional control, and increased cognitive flexibility emerged, along with significant gains in social skills and a reduction in challenging behaviors. For further details on these studies, see Table 4.

### 3.5. Pharmacological Intervention

The n = 28 studies that explored pharmacological intervention were mainly performed on MPH (n = 11/28, 39%) and ATX (n = 11/28, 39%). Only a few studies explored other pharmacological agents such as GFC (n = 4/28, 15%), clonidine (n = 1/28, 3.5%), and atypical antipsychotics (n = 1/28, 3.5%). Globally, the sample size ranged from 8 to 174 participants, and the mean duration of the intervention was 19.47 weeks (±5.5 SD; range 4–144 weeks). The main effects reported concerned all ADHD symptoms (hyperactivity, impulsivity, and inattention) by n = 18/22 (81.8%) MPH and ATX studies, n = 3/4 (75%) GFC studies, and clonidine and antipsychotic studies. The prevalent side effects were gastrointestinal disorders (13/28, 46.4%) and sleep disturbances (11/28, 39.3%).

Results of pharmacological interventions were reported based on pharmacological agents.

#### 3.5.1. Methylphenidate

MPH was evaluated by n = 11 studies. In these trials, the range of dose administered was 0.125–1.5 mg/kg/day. Treatment weeks ranged between single doses and 16 weeks, and the age range of the sample was 5–18 years, with a mean of 10.6 ± 2.0 years. See Table 2 for more details.

Nine of 11 studies (81.8%) reported improvements in hyperactivity. The remaining two studies showed benefits in processing speed [44] and emotional dysregulation [45].

Both parents and teachers described the hyperactivity reduction in n = 4/9 (44.4%) [35,37,39,40]. Stereotyped behaviors and social withdrawal were assessed in 6 (18.2%) and 7 (21.2%) MPH studies, respectively. However, only 3 studies (9.1%) reported effects on stereotypies with conflicting results, and none demonstrated a consistent impact on social withdrawal. The RUPP Autism Network conducted the first RCT on MPH [35], and subsequently Posey et al. [36] and Scahill et al. [37]. The authors, in a crossover design trial, tested three different dosages of MPH immediate release (IR): the low dose (0.125 mg/kg ×2/day + half dose at 4 p.m.), the medium dose (0.25 mg/kg ×2/day + half dose at 4 p.m.), and the higher dose (0.5 mg/kg ×2/day + half dose at 4 p.m.). Parents and teachers reported improvements in ADHD symptoms, especially in hyperactivity. Afterward, the same authors reported an open-label extension study [35] that confirms previous results. Many different AEs were reported, especially at medium and high doses, including decreased appetite, difficulty falling asleep, irritability, emotional outbursts, and stomach discomfort. Specifically, six participants discontinued during the test-dose phase and seven during the crossover phase due to intolerable side effects. Additionally, the highest dose was excluded from analysis for 16 subjects who could not tolerate it.

Likewise, Pearson et al. [40] tested three different dosages of MPH IR: in the lowest one (0.21 mg/kg + 0.14 mg/kg), improvements in hyperactivity and impulsivity were recorded only by the teachers; with the medium (0.35 mg/kg + 0.24 mg/kg) and high doses (0.48 mg/kg + 0.27 mg/kg), the same effects were also noticed by parents. At the higher dose, clinicians reported the most dramatic improvement, with amelioration in oppositional behaviors, irritability, social skills, and inappropriate speech noticed by both parents and teachers. The same authors, in a successive paper [41], reported significant improvements in sustained and selective attention and inhibition/impulsivity domains, measured by standardized tests, with linear dose/response effects for the dose range studied. Furthermore, n = 5 patients discontinued afternoon IR-MPH due to irritability (n = 5/5), decreased sleep (n = 2/5), and increased stereotyped behaviors (n = 2/5). Additionally, AEs were reported only in the highest dose, mainly loss of appetite and sleep problems [40].

Also, three different doses of MPH IR (low 0.5 mg/kg ×3/day, medium 1.0 mg/kg ×3/day, and higher 1.5 mg/kg ×3/day) were tested by Simonoff et al. [39]. The authors confirmed improvement in hyperactivity reported by both parents and teachers at all three doses. The main reported AEs were sleep difficulties, loss of appetite, and weight change, with n = 5 withdrawals due to adverse events. Among the nine studies that reported improvement in the hyperactivity domain, the study by Santosh and coll. (within-subjects study) [38], using doses of MPH IR ranging between 31.7 mg/day and 33.8 mg/day, reported enhancement in oppositional and aggressive behaviors. Moreover, giddiness, gastrointestinal symptoms, and sleep difficulties [38] were reported. No withdrawal was described. In a successive study, Golubchik et al. (open-label design) [42], using a range of doses of 10–54 mg/day of MPH IR, reported amelioration of school-related anxiety. A within-subjects design study reported no significant improvement in ADHD core symptoms on MPH IR. The authors [44] measured the efficacy of a single dose of 10 mg MPH IR on neuropsychological functions. In particular, they found improvement only in processing speed among the assessed executive functions tested after MPH IR single dose.

Only two studies used MPH extended-release (ER). Kim et al. [43] confirmed improvement in ADHD core symptoms reported by parents in both doses (0.29 mg/kg, 0.5 mg/kg), with greater effects at the highest dose. Additionally, the authors reported reduced stereotyped behaviors, inappropriate speech, social withdrawal, and irritability for medium doses. However, rebound at the end of the day, aggression, and irritability were significantly more reported for medium doses. Decreased appetite and sleep difficulties were also reported at all doses.

Finally, Ventura et al. [45], in a naturalistic study, report a significant reduction in emotion dysregulation in children and adolescents treated with MPH ER 0.8 mg/kg for 12 weeks. Side effects were reported, such as loss of appetite, abdominal discomfort, headache, irritability, anxiety, and palpitation. Two participants interrupted treatment for restlessness and increased stereotyped behaviors.

#### 3.5.2. Atomoxetine

A total of 11 studies investigated ATX, with doses ranging from 0.5 to 1.8 mg/kg/day and treatment durations between 6 and 24 weeks (average: 14.8 weeks). The sample included children aged 6–17 years (mean: 8.2 years), with a majority being male. Further details are shown in Table 2.

All the studies reported improvement in ADHD core symptoms for ATX treatment. In five studies, teacher ratings confirmed effects in the school setting [36,48,49,51,52].

Four studies reported additional benefits on ASD-related symptoms: social withdrawal improved in three studies and stereotyped behaviors in two. 

Posey et al. [36] observed improvements in irritability, social withdrawal, stereotyped behaviors, and inappropriate speech at doses of 1.2–1.4 mg/kg/day, with decreased appetite and sleep problems. according to parents’ reports. Similarly, Harfeterkamp et al. [49] confirmed benefits on stereotyped behaviors and inappropriate speech but also noted sedation, irritability, and thirst alterations, with one withdrawal due to fatigue.

Irritability was also reported by Fernandez [51], in association with somnolence and gastrointestinal symptoms, using an ATX dose of 39.79 mg/day (±12.46). A following open-label extension was conducted by Harfeterkamp [50]. The study confirmed previous results using ATX doses adjusted according to the patient’s tolerability within a range of 0.8–1.2 mg/kg/day. AEs additionally reported gastrointestinal symptoms, fatigue, and headache.

Another double-blind RCT [52] compared four types of intervention for ADHD and ASD symptoms: ATX (1.35 mg/kg/twice a day), ATX (1.38 mg/kg/twice a day) combined with Parent Training (PT), only PT, and placebo. After four weeks of treatment, ADHD symptoms did not improve, only in the placebo group. As far as the AEs, the ATX group, compared to only the PT and placebo groups, showed appetite decrease [53]. In the subsequent open-label extension study, Smith et al. [54] confirmed the efficacy of 1.16 mg/kg doses of ATX (with/without PT) on ADHD core symptoms and home-challenging behaviors. The AEs experienced were appetite decrease, gastrointestinal symptoms, headache, sleep difficulties, and fatigue. Furthermore, two pilot studies described ATX efficacy on hyperactivity and inattention/impulsivity. The first [47] is a pilot within-subject study that reported several AEs (decreased appetite, irritability, sleep problems, and mean heart rate increase) with an ATX dose of 1.19 mg/kg/day. Conversely, the double-blind, placebo-controlled crossover pilot of Arnold et al. [46] reported no significant difference among ATX and placebo groups in AEs. However, the authors included only subjects with a mental age ≥ 18 months. Furthermore, n = 6/16 subjects were taking concomitant psychotropic drugs during the study. Among these six subjects, n = 3 were assumed on add-on Risperidone (1 mg/die, 2.5 mg/die, and 3 mg/die, respectively). N = 1 was taking Aripiprazole 10 mg and Divalproex 250 mg/die, and another one assumed Sertraline 12.5 mg/die; Ziprasidone 60 mg/die; and Donepezil 5 mg/die. Finally, 1 subject was taking Buspirone 5 mg/die and Paroxetine 30 mg/die. Finally, only one study focused specifically on patients with intellectual disability. Kilincaslan et al. [55], in a retrospective study on individuals with ADHD and low-functioning ASD (age range 6–17 years), described improvements in ADHD core symptoms and social withdrawal; they also reported drowsiness, dizziness, and increased blood pressure as AEs.

#### 3.5.3. Guanfacina

Four trials evaluated the effects and tolerability of GFC. In these studies, the range of doses administered was 3–5 mg/day. The treatment duration was eight weeks. The age range of the sample was 5–14 years, with a mean of 8.75 ± 0.4 years, and male gender was prevalent. See Table 2 for other details.

A prospective open-label trial was conducted by Scahill [56] and McCracken [57]. With an 8-week treatment of GFC IR 3–5 mg/day, improvement in hyperactivity and attention was reported by parents and teachers; moreover, parents described reductions in irritability, social withdrawal, and stereotyped behavior. Additionally, irritability, sedation, and sleep disturbance have been reported as AEs. Furthermore, GFC ER was tested by RTC of Politte [59] and Scahill [58]; ADHD core symptoms, oppositional, and repetitive behaviors seem to improve with 3–4 mg/day GFC ER. Drowsiness, fatigue, decreased appetite, dry mouth, emotionality/tearfulness, irritability, anxiety, and mid-sleep awakening were reported, and n = 2 subjects withdrew due to AEs.

#### 3.5.4. Other Pharmacological Interventions

A very small study [60] explored the effect of clonidine on a sample of eight subjects, all males (age range 5.0–13.4; average age 8.1 years); the dose administered was 0.15–0.20 mg/day; modest effects on irritability and hyperactivity were described in the short-term treatment, as well as stereotyped behaviors, and no AEs were reported. Finally, a pilot, open-label, randomized, controlled trial on risperidone (RIS) and aripiprazole (ARI) [61] was conducted on a sample of 44 children (RIS n = 22, M:F = 9:2; mean 7.8 years; ARI n = 22, M:F = 9:3; mean 8.4 years). The administered doses were 3 mg/day for RIS and 15 mg/day for ARI. Both drugs improved ADHD symptoms in autistic children with comparable levels of effectiveness. As concerns tolerability, increased appetite occurred in 22.8% of participants with ARI and 50% of those taking RIS. Weight gain was observed in 18.2% of the ARI group and 36.4% of the RIS group. The authors concluded that a slightly better tolerability profile of ARI. Details in Table 2.

### 3.6. Response Moderators

Of the total 32 studies included in our review, only 10 specifically investigated potential moderating agents that could influence treatment outcomes. Of these, 6/10 studies were inherent in MPH, 3/10 studies were inherent in ATX, and only 1/10 were in GFC.

In the subset of studies using MPH, 6 of 11 (54.5%) assessed whether factors such as age [35,36,43], sex [41], IQ level [35,36,39], body weight [35,36], or diagnostic category [35,36,39,44] moderated treatment response. Five of these studies found elements acting as modulators on treatment response. In contrast to these studies, Peled et al. [44] provided unique insight by reporting that the co-occurrence of ADHD and ASD negatively impacted treatment response. Specifically, children diagnosed exclusively with ADHD showed significant improvements in attention, processing speed, impulsivity, and hyperactivity following MPH treatment. In contrast, among children with comorbid ADHD and ASD, MPH was associated with improvements in processing speed only, without notable effects on attention, impulsivity, or hyperactivity.

Similarly, among the ATX studies, only 3 out of 11 (27.7%) examined diagnosis, age, IQ, and body weight [48,52,54] as potential moderators of treatment response. Notably, none of these studies identified a moderator whose influence was statistically significant on treatment outcomes.

Furthermore, the genetic influence on treatment response was evaluated in only one study. Specifically, McCracken et al. [57] investigated the role of MDR1 gene polymorphisms as moderators of response to GFC treatment. This study suggested that a single-nucleotide polymorphism variant of the MDR1 gene may influence the drug’s efficacy, mainly concerning hyperactive-impulsive symptoms. Particularly, subjects homozygous for the minor allele T of the C34535T MDR1 variant (T=T) showed an improvement reduced by three times compared with other genotypes (C=T and C=C) (Table 5).

### 3.7. Quality Assessment

According to the AHRQ criteria, the quality assessment of the randomized controlled trials (RCTs) included in this review revealed that four studies were classified as having “fair” methodological quality, while only two reached the “good” quality threshold. Random sequence generation was explicitly described in eight studies [37,39,48,49,52,59,60,67], and allocation concealment procedures were reported in 11 trials [37,39,40,41,43,48,49,52,60,66,67]. Sample size estimation through power analysis was addressed in all RCTs, with two studies offering particularly detailed descriptions [37,39]. All trials adopted a double-blind design, except for two [66,67]. Regarding attrition bias, five RCTs were assessed as having a low risk in this domain [37,39,48,49,59], whereas the remaining studies did not provide sufficient data to make a definitive judgment. Moreover, eight studies gave a comprehensive account of patient flow, including the number screened, exclusions with justifications, and losses to follow-up [37,39,40,48,49,52,66,67]. However, only 12 RCTs included a flow diagram illustrating the participant selection process [37,39,40,41,43,48,49,50,52,54,66,67]. Trial protocol registration in a public database, as recommended by the CONSORT 2010 statement [70], was reported in all but two studies [66,67].

For non-randomized studies evaluated using the Newcastle-Ottawa Scale (NOS), five were classified as “poor quality” and eight as “fair quality”. Among these, all but two were prospective observational studies; the remaining two were retrospective observational analyses [38,55]. The exposure cohort was considered representative in eleven studies, and the methods of exposure assessment—relying on structured interviews or objective sources—were adequately reported in the same number of studies. However, only four studies detailed the criteria used to exclude individuals from the unexposed cohorts. Outcome assessments for variables not present at baseline were clearly described in eleven studies. Follow-up duration ranged from three to six months and was deemed appropriate in seven studies. Notably, none of the included studies achieved comparability between cohorts through design or statistical adjustment for relevant confounders. Overall, the non-randomized studies demonstrated a consistently high risk of bias. Risk of bias assessments across studies are summarized in Table 6. Agreement between the two independent reviewers (GL, AA) was high.

## 4. Discussion

This review examined the current literature aimed at providing evidence on the reported outcomes and safety of the treatments for children and adolescents with co-occurring ASD and ADHD.

To our knowledge, our study represents the most comprehensive synthesis to date of interventions targeting this comorbid population. We focused on several clinically relevant variables: sample characteristics, outcome measures, reported outcomes on core symptoms, adverse events, and tolerability. Furthermore, we also investigated important effect modifiers in this neglected patient population.

Indeed, ADHD and ASD are neurodevelopmental conditions that often coexist and share neurocognitive pathways [71,72]. In both disorders, it is possible to find attention deficits [73,74] and behavioral problems, such as impulsivity and hyperactivity [73]. Additionally, children with ASD and ADHD often have deficits in social skills, including difficulties in interpreting social cues, sustaining conversations, and forming peer relationships [75,76]. Despite these overlaps, these disorders have often been studied separately. The main reason is that dual/concomitant diagnoses of ADHD and ASD were not formally allowed until the revision of the DSM in 2013, or DSM-5 [1]. Consequently, recommended treatments for ASD and ADHD have been separately studied. Furthermore, the substantial heterogeneity among individuals with both disorders and the lack of pharmacological treatments with direct effects on ASD core symptoms have further slowed down research in this field [23].

Although stimulant medications are commonly prescribed to children with co-occurring ASD and ADHD, relatively few controlled studies have evaluated their behavioral and cognitive effects in this population [41]. Future research should include both individuals with single diagnoses and those with dual diagnoses to better reflect real-world clinical populations. The heterogeneity of samples in terms of age, IQ, and ethnicity may affect the generalizability of the findings. In terms of sample size, this parameter varies significantly (from 8 to 128), which may impact the statistical robustness of the findings. Moreover, the heterogeneity in sample sizes could affect the study’s power and the ability to draw generalizable conclusions. Participants’ ages range from four to 18 years (mean age 9.5 ± 1.7 years), suggesting a focus on children rather than adolescents. Additionally, the majority (63.33%) of participants are White, potentially limiting ethnic representativeness. A male predominance was observed, consistent with ADHD and ASD prevalence but raising concerns about the representation of females. Only 37.5% of studies involved samples with IQ > 70, 12.5% with IQ < 70, and 43.7% included mixed IQ samples. The limited number of studies on children with an IQ < 70 suggests a gap in research on populations with intellectual disabilities. Two studies did not report participants’ IQ, highlighting a potential methodological limitation.

Outcome measures were highly heterogeneous, limiting comparability and preventing meta-analyses. Most studies relied on parent-reported questionnaires, particularly the Aberrant Behavior Checklist (46.9%), the Conners’ Parent Rating Scale (31.2%), and the SNAP-IV (21.9%). While informative, parent reports are susceptible to bias (e.g., subjective perception, recall bias). Clinical observation was included in most studies, with the CGI scale being the most frequently used measure (59.4%). Although useful for assessing global improvement, CGI may lack the specificity of more detailed behavioral tools.

On the effectiveness of ADHD core symptoms, all medications demonstrated greater short-term efficacy than Specifically, among the drugs with the largest number of included studies, 81.8% of MPH studies and 100% of ATX studies reported improvement in the hyperactivity domain and attention impairment. Regarding ASD-specific symptoms, only a few studies have evaluated and shown a positive effect on stereotyped behaviors and social withdrawal. Most evidence is regarding a decrease in social withdrawal (3/4 studies) and stereotyped behaviors (2/4). On the other hand, only 2/7 studies on MPH reported a decrease in social withdrawal, and 1/7 in stereotyped behaviors.

In terms of tolerability, MPH showed a lower rate of treatment discontinuation due to adverse events (7.2%) compared to ATX (14.7%) and guanfacine (2.3%). However, these differences should be interpreted in light of the methodological variability across studies, particularly in how adverse events were reported and assessed. Appetite, sleep difficulties, and gastrointestinal symptoms were the most frequently reported adverse events, all of which ended after discontinuation of the drug.

Comparative tolerability between MPH and ATX remains an important issue. A notable finding, which requires confirmation in head-to-head RCTs, is the similar effectiveness of MPH and ATX for core ADHD symptoms. However, variation in dosing and outcome measures across studies limits the generalizability of this observation, highlighting the need for larger-sample, structured RCTs directly comparing these two pharmacological treatments.

Overall, MPH appears to be an effective first-line treatment for ADHD symptoms in children with ASD, despite a somewhat lower response rate (50–60%) compared to ADHD-only populations (75%) [35]. Symptom reduction is typically more pronounced for hyperactivity-impulsivity than for inattention [37]. Although limited, some evidence suggests that MPH may also improve emotional regulation, a frequent challenge in both ASD and ADHD populations [45].

MPH has demonstrated a favorable safety profile, with no reported exacerbation of repetitive or oppositional behaviors. While a slight increase in seizure incidence has been noted during the initial treatment phase [77], overall seizure risk remains low [36]. ATX represents a viable non-stimulant alternative, especially in cases of comorbid conditions such as anxiety, tics, or emotional dysregulation [78].

It may also benefit core ASD symptoms, including stereotypies and deficits in cognitive flexibility, particularly in higher-functioning individuals or those with poor verbal skills [52]. Nevertheless, its impact on global functioning remains unclear [36,49,77,78,79,80]. Comparing the effectiveness of MPH and ATX is a challenge because of the heterogeneity of the outcome measures [81]. Another critical factor influencing treatment outcomes is the variability in the dosing of MPH and ATX across different studies. Studies that fell outside these recommended dosing ranges might have contributed to the observed variability in treatment responses and adverse events. Future research should aim for more standardized dosing protocols to enhance the comparability of results and provide clearer guidance for clinical practice. Overall, a reasonable recommendation could be the use of MPH as the first choice and of ATX if coexisting symptoms, such as tics, mania, and suicidal ideation, exacerbate the core symptoms [82,83,84,85].

Clonidine and guanfacine may be considered second-line treatments, particularly in children with irritability, mood instability, or partial responses to first-line agents [23,27,60]. While less frequently studied, they have shown promise in managing a range of maladaptive behaviors.

Antipsychotics, especially second-generation agents like risperidone (RIS) and aripiprazole (ARI), are widely used in clinical practice to address irritability and aggression in children with ASD [86,87]. However, due to the lack of robust evidence on their effects on ADHD symptoms, their use should be limited to specific therapeutic goals (e.g., severe aggression) rather than core ADHD management.

Based on these findings, we propose the following clinical considerations. While further research is needed, current evidence supports a stepwise clinical approach for the management of children and adolescents with co-occurring ASD and ADHD.

MPH may represent a first-line treatment, particularly in the absence of contraindications and when hyperactivity-impulsivity predominates. This recommendation is supported by the largest number of studies included in this review and consistent evidence of therapeutic effect.

ATX can be considered in cases involving emotional dysregulation, anxiety, poor sleep, or when stimulants are not well tolerated. Evidence supports its clinical benefit in both ADHD core symptoms and, in some cases, ASD-related symptoms such as social withdrawal and stereotyped behavior.

Guanfacine and clonidine are viable second-line options, especially in children with irritability, mood instability, or partial response to first-line treatments.

SGAs should not be used to target ADHD or ASD core symptoms but may be warranted in cases of severe irritability or aggression, particularly in individuals with ASD. Overall, treatment should be individualized based on the symptom profile, comorbidities, tolerability, and developmental level.

Despite the clinical importance of non-pharmacological interventions, only four studies focused on these approaches in individuals with ASD and ADHD, underscoring a major gap in the literature.

Although behavioral therapy, parent training, cognitive training, and social skills interventions are well-established in ADHD or ASD populations separately, their effects in comorbid cases remain largely unexplored [26,76,88,89,90]. Three studies evaluated the employ of technology-assisted interventions, which show promise for enhancing cognitive and behavioral outcomes. However, further research is needed to determine their long-term effectiveness and their applicability. Overall, although the studies provide preliminary evidence supporting the potential benefits of non-pharmacological interventions in children with ASD and ADHD, the limited sample size and methodological constraints underscore the need for larger studies to establish the therapeutic benefit and broader applicability of these treatments.

The lack of evidence limits clinicians’ ability to provide comprehensive, evidence-based recommendations for patients who may not tolerate or benefit from pharmacotherapy. Addressing this gap is critical to providing a more comprehensive and individualized treatment approach, ensuring that children with ADHD and ASD receive interventions that go beyond medication and support their broader cognitive, emotional, and social development.

Finally, treatment response moderators were investigated in only 10 out of 32 included studies.

Most analyses focused on MPH (6 studies) and ATX (3 studies), with only one study investigating guanfacine, highlighting the need for broader analysis across different pharmacological treatments. Specifically, significant moderators were identified in five of six MPH studies, suggesting that individual characteristics may affect treatment outcomes. No significant moderators were identified in ATX studies, raising questions about either its broader applicability or the need for more refined analyses. Finally, only one study [46] explored genetic moderators, specifically the MDR1 gene polymorphism in guanfacine treatment, suggesting that the MDR1 C34535T polymorphism may impact treatment response. This highlights the potential role of pharmacogenetics in ADHD and ASD treatment, which remains underexplored.

## 5. Limitations

This review has several limitations. Despite our efforts to include all available studies on the topic, we cannot exclude that there is some missing information due to the exclusion of potentially relevant studies in languages other than English. In addition, a potential publication bias cannot be excluded, especially considering the scarcity of non-pharmacological studies and the likelihood that negative or null results remain unpublished. Due to the heterogeneity of study designs and outcomes, funnel plots could not be constructed to formally assess this bias.

Moreover, the representation of specific subgroups (e.g., age, gender, level of intellectual functioning) varies across studies, potentially limiting the generalizability of our findings to all individuals with ASD and ADHD. Also, the diverse assessment methods used in the included studies (e.g., questionnaires, clinical interviews, direct observations) could be a source of biases in detecting and characterizing the ADHD-ASD phenotype. Finally, the literature related to non-pharmacological interventions in individuals with ASD + ADHD remains very limited and underdeveloped, making it difficult to draw definitive conclusions.

## 6. Future Direction

The overlap between ADHD and ASD often leads to worse clinical outcomes and reduced quality of life [91], highlighting the importance of interventions, not only in effectively addressing symptomatology but also in prioritizing safety and tolerability [92]. A novel and increasingly relevant approach to managing ADHD symptoms in individuals with ASD challenges the traditional concept of comorbidity, instead proposing that attentional deficits be recognized as intrinsic to the clinical profile of ASD [93]. This reconceptualization further reinforces the need to develop treatment algorithms that appropriately address ADHD symptoms in the ASD population. Future studies should prioritize larger and more demographically balanced sample sizes to improve statistical power and enhance the generalizability of findings. Greater inclusion of ethnically different populations is essential to mitigate potential sampling biases and ensure broader applicability of the results.

Moreover, there is a pressing need for studies focusing specifically on adolescents, a group that is currently underrepresented despite being at a critical stage of neurodevelopment, to better understand symptoms and treatment responses in that specific developmental window. Longitudinal design studies are particularly needed to assess long-term treatment effects and to elucidate developmental trajectories over time. Populations with intellectual disabilities remain notably underrepresented in the literature and deserve greater research attention. Furthermore, a more detailed stratification of participants based on cognitive abilities and symptom severity could lead to more tailored treatment approaches.

Regarding outcome measures, the current reliance on heterogeneous assessment tools likely contributes to variability in reported treatment effects. Therefore, future studies should aim for greater methodological consistency in assessment tools, ideally by employing standardized instruments that integrate both structured clinical observations and validated questionnaires. The combined use of objective and subjective measures could enhance the reliability and validity of symptom Non-pharmacological interventions are recognized as essential components of comprehensive care for ASD and ADHD populations. Additionally, nutraceuticals and dietary supplements have shown potential in supporting symptom management in these conditions [94]. However, the current evidence base for their efficacy in comorbid populations remains limited. There is a clear need to expand research in this area to determine which treatment approaches are most effective and beneficial for specific patient profiles.

Finally, RCT studies for directly comparing ATX and MPH are needed to clarify their relative efficacy and tolerability, particularly in patients with co-occurring ADHD and ASD versus those with a single diagnosis. The current paucity of studies examining moderators of treatment response limits progress toward personalized medicine. Future investigations should prioritize the identification of such moderators, especially genetic factors that may influence drug metabolism and treatment efficacy. Finally, elucidating the shared pathophysiological mechanisms between ADHD and ASD may facilitate the design of more targeted interventions. Addressing these research gaps is essential for advancing clinical practice and improving outcomes for individuals with co-occurring ASD and ADHD.

## Figures and Tables

**Table 1:** Sample characteristics of the included studies.

Reference	Study Design	Sample Size	Ethnicity %	Sex Ratio M:F	Age Range in Years (Mean ± SD)	IQ, Mean (SD) or % of Individuals with Intellectualdisability
RUPP., 2005 [35]; Posey et al., 2006 [36]; Scahill et al., 2017 [37]	randomized crossover trial	72	73.3% White/Non-Hispanic, 15% Black or African American, 6.7% Asian, 5% Hispanic	9:1	5–14 (7.4 ± 2.1)	IQ 40% ≥ 70 50% < 70 10% missing data
RUPP., 2005 [35]	OPEN-LABEL extention study	35	//	//	5–14 (7.4 ± 2.1)	//
Santosh et al., 2006 [38]	Retrospective	ADHD: 113	N.R.	4:1	NR (13.1 ± 3.18)	5.65%
		ASD + ADHD: 61			NR (12.4 ± 3.01)	7.32%
	Prospective, quasiexperimental, within-subjects	ADHD: 25	NR	4.5:1	NR (11.6 ± 2.5)	95.2 (16.1)
		ASD + ADHD: 27		9:1	NR (10.6 ± 2.7)	84.3 (25.1)
Simonoff et al., 2013 [39]	randomized controlled double-blind trial	61	N.R.	1:0.5	7–15 (11.5 ± 2.3)	53 (10.5)
		61		2.8:1	7–15 (10.9 ± 2.4)	54 (9.6)
Pearson et al., 2013; 2020 [40,41]	Single-blind, within-subject, dose crossover, placebo-controlled design	24	75% Caucasian, 16.6% African-American, 4.17% Asian, 4.17% multiple races	3.8:1	7.1–12.7 (8.8 ± 1.6)	85.0 (16.8)
Golubchik et al., 2017 [42]	open-label design	12	N.R.	4.5:1	8–18 (11.3 ± 2.0)	≥IQ 70
Kim et al., 2017 [43]	Pilot randomized study	Low dose group: 9	55.56% Caucasian, 18.52% multiple races, 14.81% NR, 7.41% other, 3.70% Asian	9:1	5–14(9.33 ± 2.92)	NR
		medium dose group: 18		9:1	5–15 (9.11 ± 3.12)	NR
Peled et al., 2019 [44]	Within subject	40	N.R.	9:1	6–18 (9.0 ± N.R.)	IQ in the normal range
Ventura et al., 2022 [45]	Naturalistic study	ASD + ADHD: 29	Caucasian 100%	4.5:1	6–18 (13.3 ± NR)	96.3 (NR)
		ADHD: 41		1:0	6–18 (13.4 ± NR)	97.2 (NR)
Arnold et al., 2006 [46]	double-blind, placebo-controlled, crossover pilot study	16†	81.3% White,12.5% African, 6.2% Asian	3:1	5–15 (9.26 ± 2.93)	mental age ≥ 18 months
Troost et al., 2006 [47]	Pilot, within subject study	12	N.R.	4.5:1	6–14 (10.2 ± 2.8)	≥IQ 70
Posey et al., 2006 [36]	prospective, open label study	16	68.75% white 12.5% African american 12.5% Hispanic/Latino; 6.25% african american/white	4.5:1	6–14 (7.7 ± 2.2)	93.9 ± 18.0
Harfeterkamp et al. 2012; 2014 [48,49]	double-blind RCT	48	100% White, 0% African	9:1	6–16 (9.9 ± 2.7)	91.0 (16.4)
		49	98% White, 2% African	9:2	6–17 (10 ± 2.9)	94.6 (17.7)
Harfeterkamp et al. 2013 [50]	open-label extension study	42	N.R.	9:1	6–16 (10.0 ± 2.8)	93.1 (17.3)
		46		4.5:1	6–17 (10.0 ± 3.0)	
Fernandez-Jaen et al., 2013 [51]	prospective, open label study	24†	N.R.	3:2	5–17 (8.8 ± 3.38)	80%
Handen et al. 2015 [52]; Tumuluru et al., 2017 [53]	double-blind RCT	32	87.5 Caucasian, 3.1% African	9:0	5–14.11 (8.0 ± 1.9)	83.3 (21.6)
		32	84.4% Caucasian, 6.3% African	4.5:1	5–14.11 (8.6 ± 2.3)	78.7 (25.9)
		32	81.3% Caucasian, 6.3% African	4.5:1	5–14.11 (7.7 ± 1.5)	77.9 (25.7)
		32	75.0% Caucasian, 15.6% African	9:1	5–14.11 (8.2 ± 2.4)	86.7 (23.7)
Smith et al., 2016 [54]	open-label extension study	28	85.7% Caucasian, 3.6% African American, 0% Other, 10.7% Multiracial	5:1	5–14.11 (8.0 ± 1.8)	85.4 ± 22.9
		15	60.0% Caucasian, 20.0% African American, 6.7% Other, 13.3% Multiracial	2:1	5–14.11 (8.1 ± 2.0)	74.3 ± 26.6
		19	79.0% Caucasian, 5.3% African American, 5.3% Other, 10.5% Multiracial	18:1	5–14.11 (7.7 ± 1.3)	82.9 ± 24.1
		22	81.8% Caucasian, 13.6% African American, 0% Other, 4.6% Multiracial	3:1	5–14.11 (8.4 ± 2.5)	89.4 ± 22.9
Kilincaslan et al., 2016 [55]	naturalistic retrospective study	37	N.R.	3:1	6–17 (10.16 ± 3.60)	<70; mild 27% moderate 46% severe 27%
Scahill et al., 2007 [56]; McCracken et al., 2010 [57]	Prospective Open-label Trial	25	72% caucasian, 24% black, 4% Hispanic	11.5.1	9.03 (±3.14)	31.56%
Scalhill et al., 2015 [58]; Politte et al., 2018 [59]	randomized, placebo-controlled trial	30	56.67% white, 23.3% black, 13.33% asian, 3.33% pacific islander, 3.33% mixer	9:1	5–14 (8.5 ± 2.25)	36.7%
		32	68.75% white, 12.50% black, 3.13% asian, 3.13% pacific islander, 9.38% mixer	4.5:1		33.0%
Jaselskis et al., 1992 [60]	double blind placebo controlled crossover study	8	N.R.	8	5–13.4 (8.1 ± 2.8)	30–75 (59 ± 16)
Lamberti et al., 2016 [61]	Pilot, Open-Label, Randomized Controlled Study	22	NR	4.5:1	6–13 (7.8 ± 2.3)	≥55
		22	NR	3:1	6–13 (8.4 ± 2.9)	

ADHD = attention deficit hyperactivity disorder; ASD = Autism spectrum disorder; N.R = Not Reported; IQ = Intelligence Quotient.

**Table 2:** Main characteristics and results of included pharmacological studies.

Reference	Study Design	Sample Size	Investigated Domains	Outcome Measures	Pharmacological Treatment	Mean or Range Dose	Treatment Duration	Efficacy
RUPP., 2005 [35]; Posey et al., 2006 [36]; Scahill et al., 2017 [37]	Randomized crossover trial	72	Hyperactivity, irritability, social withdrawal, stereotyped behaviors, inappropriate speech, distractibility, impulsivity, challenging behaviours	ABC (parent and teacher raters), CGI (RUPP 2005 [35], Scahill 2017 [37]); SNAP-IV, CYBOCS-PDD, (Posey 2006 [36]); PTPs (Scahill 2017 [37]);	Placebo	//	1 week for each dose	//
					MPH IR	~0.125 mg/kg ×2/day + half dose at 4 P.M.		Hyperactivity (both parents and teachers), inattention
						~0.25 mg/kg ×2/day + half dose at 4 P.M.		Hyperactivity (both parents and teachers); ADHD-related problems targeted by parents
						~0.5 mg/kg ×2/day + half dose at 4 P.M.		Hyperactivity (both parents and teachers); ADHD-related problems targeted by parents. The greatest improvement was recorded at the highest dose
RUPP., 2005 [35]	OPEN LABEL extension study	35	hyperactivity, irritability, social withdrawal, stereotyped behaviors, inappropriate speech	ABC (parent and teacher raters)	MPH IR	adjusted on clinical judgment	8 weeks	Hyperactivity (both parents and teachers)
Santosh et al., 2006 [38]	Retrospective	ADHD: 113	therapeutic effects on degree of illness	CGI and TEI	MPH	MPH 30 mg/day in 3 divided doses	8 weeks	The clinical response was not statistically different between the groups
		ASD + ADHD: 61				MPH 30 mg/day in 3 divided doses		
	Prospective, quasiexperimental, within-subjects	ADHD: 25	hyperactivity, impulsivity, inattention, oppositionality, aggressivity, intermittent explosive rage, antisocial behav., tics, social communication, language, clumsiness, repetitive behav, circumscribed interests, cognitive rigidity, self-injury, stereotypies, hypersensitivity, lack of remorse, obsessions, low mood, depressive ideation, warries, panic, fears, labile mood, somatic symp., eating probl, sleep probl., bed wetting, hallucination, seizures	PONS-C, CGI	MPH	mean dose 31.7 mg/day	8 weeks	hyperactivity, impulsivity, inattention, oppositionality, aggression and intermittent explosive rage
		ASD + ADHD: 27				mean dose 33.8 mg/day		hyperactivity, impulsivity, inattention, oppositionality, aggression and intermittent explosive rage
Simonoff, et al., 2012 [39]	randomized controlled double-blind trial	61	inattention, hyperactivity, impulsivity, irritability, social withdrawal, stereotyped behaviors, inappropriate speech	CPRS-R, CTRS-R, ABC, CGI-I	Placebo	//	16 weeks (at least 1 week of dose titration for each dose; after the optimal dose for each participant was prescribed for the remaining of 16 weeks)	//
		61			MPH IR	0.5 mg/kg ×3/day		hyperactivity and Conners ADHD index (both parents and teachers); 40% improved at CGI
						1.0 mg/kg ×3/day		
						1.5 mg/kg ×3/day		
Pearson et al., 2013; 2020 [40,41]	Single-blind, within-subject, dose crossover, placebo-controlled design	24	inattention, hyperactivity, impulsivity, emotional lability, irritability, oppositional, social withdrawal, social skills, social communication, stereotypy, and inappropriate speech (Pearson D.A. et al., 2013 [40]). Sustained attention, selective attention, impulsivity/inhibition (Pearson D.A. et al., 2020 [41])	CPRS-R, CTRS-R, SNAP-IV, ACTeRS, ABC, SCQ, VAS, CGI (Pearson D.A. et al., 2013 [40]); CPT, SCT, PSIT, GDS, MFFT, SST (Pearson D.A. et al., 2020 [41])	Placebo	//	1 week for each dose	//
					MPH (ER morning + IR afternoon doses)	LA 0.21 mg/kg + IR 0.14 mg/kg		teachers detected significant improvements in hyperactivity and impulsivity (Pearson D.A. et al., 2013 [40]); gain in sustained attention, selective attention both auditive and visive, impulsivity/inhibition
						0.35 mg/kg + 0.24 mg/kg		Both parents and teachers: hyperactivity, impulsivity, social skills (Pearson D.A. et al., 2013 [40]); gain in sustained attention, selective attention both auditive and visive, impulsivity/inhibition
						0.48 mg/kg + 0.27 mg/kg		Both parents and teachers: oppositional behavior, hyperactivity, impulsivity, attention, irritability, inappropriate speech, and social skills. These effects were greater than the medium dose. Clinicians reported the most dramatic improvement. (Pearson D.A. et al., 2013 [40]); greater improvement in sustained attention at the highest dose, selective attention both auditive and visive, impulsivity/inhibition.
Golubchik et al., 2017 [42]	open-label design	12	inattention, hyperactivity, impulsivity, anxiety and depression symptoms	ADHD-RS, SCARED, CDI	MPH	10–54 mg/day	12 weeks	attention-deficit/hyperactivity symptoms, school-related anxiety
Kim et al., 2017 [43]	Pilot randomized study	Low dose group: 9	inattention, hyperactivity, impulsivity, irritability, social withdrawal, inappropriate speech, stereotyped behaviors.	ADHD-RS-INV, CGI-I, ABC	MPH ER	0.29 mg/kg	6 weeks	attention, hyperactivity, impulsivity and clinical global improvement for low and medium doses, with greater improvement at the highest dose. Additionally, irritability, lethargy, inappropriate speech, and stereotyped behaviors only for medium dose.
		medium dose group: 18				0.5 mg/kg		
Peled et al., 2019 [44]	Within subject	40	inattention, hyperactivity, processing speed, impulsivity	MOXO-CPT	MPH IR	10 mg i.r.	single dose	Processing speed
Ventura et al., 2022 [45]	Naturalistic study	ASD + ADHD: 29	Emotional disregulation	CBCL	MPH ER	0.8 mg/kg	12 weeks	significant reduction in emotion dysregulation
		ADHD: 41				0.78 mg/kg	12 weeks	significant reduction in emotion dysregulation
Arnold et al., 2006 [46]	double-blind, placebo-controlled, crossover pilot study	16†	inattention, hyperactivity, impulsivity, irritability, social withdrawal, stereotyped behaviors, inappropriate speech; Self-injury, Compulsions, Rituals, Restrictive	ABC hyperactivity subscale, RBSR, CGI-S, CGI-I, CPT, DMST, ACT	Placebo	//	6 weeks	//
					ATX	1.4 mg/kg/day in split dose (maximum daily dose)		hyperactivity, impulsivity, social withdrawall
Troost et al., 2006 [47]	Pilot, within subject study	12	inattention, hyperactivity, impulsivity, oppositional, irritability, social withdrawal, stereotypy, inappropriate speech	ADHD-RS IV, CPRS-R, ABC; CGI-ADHD-S	ATX	0.5–1.8 mg/kg/day (divided into two doses based on patient-preference)	10 weeks	Hyperactivity, attention
Posey et al., 2006 [36]	prospective, open label study	16	inattention, hyperactivity, impulsivity, irritability, social withdrawal, stereotype, inappropriate speech, social responsiveness.	SNAP-IV reported both by parents and teachers, ABC, CGI-I, SRS	ATX	1.2–1.4 mg/kg/day	8 weeks	adhd total score reported both by parents and teachers; irritability, social withdrawal, stereotype, inappropriate speech reported by parent
Harfeterkamp et al. 2012; 2014 [48,49]	double-blind RCT	48	inattention, hyperactivity, impulsivity (Harfeterkamp M., et al. 2012 [48]); irritability, social withdrawal, stereotypic behavior, inappropriate speech, subtle social, communicative, and repetitive behaviors (Harfeterkamp M., et al. 2014 [49]);	ADHD RS-IV, CTRS-R:S, CGI-ADHD-S (Harfeterkamp M., et al. 2012 [48]); ABC, CSBQ (Harfeterkamp M., et al. 2014 [49])	ATX	1.2 mg/kg/day	8 weeks	Hyperactivity, inattention, and impulsivity, reported both by parents and teachers (Harfeterkamp M., et al. 2012 and 2014 [48,49]); inappropriate speech, and stereotypic behavior, fear of changes (Harfeterkamp M., et al. 2014 [49])
		49			Placebo	//		//
Harfeterkamp et al. 2013 [50]	open-label exstension study	42	inattention, hyperactivity, impulsivity	ADHD-RS, CGI	ATX	0.8–1.2 mg/kg/day, based on tolerability	20 weeks	Hyperactivity, inattention, impulsivity
		46						
Fernandez-Jaen et al., 2013 [51]	prospective, open label study	24†	inattention, hyperactivity, impulsivity, conduct problems	ADHD RS-IV, CPRS-R, CTRS-R:S, CGI-I	ATX	39.79 mg/day (±12.46)	16 weeks	Hyperactivity, inattention, impulsivity, reported both by parents and teachers
Handen et al. 2015 [52]; Tumuluru, et al., 2017 [53]	double-blind RCT	32	inattention, hyperactivity, impulsivity; non-compliance (parents and teachers raters); irritability, social withdrawal, stereotypy, inappropriate speech (Handen B. L., et al. 2015 [52])	SNAP IV and ABC (parent and teacher rater), CGI-ADHD, HSQ, SSQ (Handen B. L., et al. 2015 [52])	ATX + PT	1.35 mg/kg/split twice daily	10 weeks (6 w tritation + 4 w manteinment)	Hyperactivity, inattention, impulsivity, reported by parents; inappropriate speech, home non-compliance
		32			ATX	1.38 mg/kg/split twice daily		Hyperactivity, inattention, impulsivity, reported by parents; home non-compliance
		32			Placebo + PT	//		Hyperactivity, inattention, impulsivity, irritability, reported by parents; inappropriate speech (by parents and teachers), school non-compliance.
		32			Placebo	//		None
Smith et al., 2016 [54]	open-label extension study	28	inattention, hyperactivity, impulsivity; non-compliance (parents), irritability, social withdrawal, stereotypy, inappropriate speech	SNAP IV and ABC (parent rater), CGI-ADHD, HSQ	ATX	1.16 mg/kg	24 weeks	Hyperactivity, inattention, impulsivity, home non-compliance
		15			Placebo	//		//
		19			ATX + PT	1.16 mg/kg		Hyperactivity, inattention, impulsivity, home non-compliance
		22			Placebo + PT	//		no significant difference
Kilincaslan et al., 2016 [55]	naturalistic retrospective study	37	inattention, hyperactivity, impulsivity, irritability, social withdrawal, stereotyped behaviors, inappropriate speech;	ADHD-RS, ABC, CGI, CARS	ATX	1.20 ± 0.11 mg/kg/day	12 weeks	Hyperactivity, inattention, impulsivity, social withdrawal
Scahill et al. 2007 [56]; McCracken et al. 2010 [57]	Prospective Open-label Trial	25	inattention, hyperactivity, irritability, social withdrawal, stereotypy, inappropriate speech	ABC, SNAPIV (both parents and teachers), CGI-I (Scahill, L et al. 2006) [56]	Guanfacine	3–5 mg/day	8-weeks	Hyperactivity, Inattention (parents and teachers raters), Irritability, Social withdrawal, Stereotypy (parents raters)
Scalhill, et al. 2015 [58]; Politte et al. 2018 [59]	randomized, placebo-controlled trial	30	inattention, hyperactivity, irritability, social withdrawal, stereotypy, inappropriate speech, working memory, motor planning (Scailhill, L. et al. 2015 [58]); oppositional behavior, non-compliance, anxiety, repetitive behavior, and sleep disturbance (Politte, L. et al. 2018 [59])	ABC, ADHD-RS, CGI-I, RPDR, NEPSY II (Scailhill, L. et al. 2015 [58]), HSQM, CASI, CYBOCS-ASD, CSHQ (Politte, L. et al. 2018 [59])	ER Guanfacine	3–4 mg/day	8 weeks	Hyperactivity, inattention, stereotypy, inappropriate speech (Scailhill, L. et al. 2015 [58]); hyperactivity, oppositional and repetitive behavior (Politte, L. et al. 2018 [59])
		32			Placebo	//		//
Jaselskis et al., 1992 [60]	double blind placebo controlled crossover study	8	ADHD Index, inattention, hyperactivity, impulsivity, irritability, social withdrawal, stereotypy, inappropriate speech, oppositivity, non-compliance	CPRS-R, CTRS-R, ACTeRS, ABC (parent and teachers raters), HSS, CGI-I	Placebo	//	6 weeks	//
					Clonidine	0.15–0.20 mg/day		ADHD Index (parents raters), hyperactivity, irritability, stereotypy, inappropriate speech, oppositional behaviours (teachers raters)
Lamberti et al., 2016 [61]	Pilot, Open-Label, Randomized Controlled Study	22	general functioning, inattention, hyperactivity, impulsivity, oppositional, ADHD Index, severity of illness	C-GAS, CGI-S, ADHD R-S, CPRS-R:S	Risperidone	3 mg/day	12 weeks	ADHD-RS index, hyperactivity, illness severity
							24 weeks	ADHD-RS index, hyperactivity, illness severity
		22			Aripiprazole	15 mg/day	12 weeks	ADHD-RS index, hyperactivity, inattention, illness severity, increased global functioning
							24 weeks	ADHD-RS index, hyperactivity, inattention, illness severity, increased global functioning

**Pharmacological agent/group legend**: MPH = Methylphenidate; ATX = atomoxetine; IR = Immediate Release; ER = Extended Release; PT = Parent Training; **Legend Test**: Moxo-CPT = MOXO-Continuous Performance Test; CPRS-R = Conners Parent Rating Scale-Revised; CTRS-R = Conners Teacher Rating Scale-Revised; SNAP-IV = Swanson, Noland, and Pelham Scale; ACTeRS = ADHD Comprehensive Teacher Rating Scale; ABC = Aberrant Behavior Checklist; SCQ = Social Communication Questionnaire; VAS = Visual Analog Scale; CGI-I = Clinical Global Impressions-Improvement; CGI-S = Clinical Global Impressions-Severity; CPT = Continuous performance test; SCT = Speeded classification task; PSIT = Pediatric Speech Intelligibility Test; GDS = Gordon Diagnostic System; MFFT = Matching Familiar Figures Test; SST = Stop signal task; CYBOCS-PDD = Children’s Yale-Brown Obsessive Compulsive Scales for PDD; PTPs = Parent Target Problems; CBCL = Child Behavior Checklist; ADHD RS-INV = ADHD Rating Scale, Investigator Version; TEI = Therapeutic Efficacy Index; PONS-C = Profile of Neuropsychiatric Symptoms; SCARED = Screen for Child Anxiety Related Emotional Disorders; CDI = Children’s Depression Inventory; RBSR = Repetitive Behavior Scale-Revised; CPT = Continuous Performance Task; DMST = Delayed Match-to-Sample Task; ACT = Analogue Classroom Task; HSQ = Home Situations Questionnaire; SSQ = Scholl Situations Questionnaire; SRS = Social Responsiveness Scale; CSBQ = Children’s Social Behavior Questionnaire; CARS = Childhood Autism Rating Scale; ACTr-Rs = Attention Deficit Disorder with Hyperactivity Comprensive Teacher’s Rating Scale; ; C-GAS = Children’s Global Assessment Scale; ADHD Rating Scale-IV.

**Table 3:** Safety measures and adverse events of included studies.

Reference	Pharmacological Agent	Mean or Range Dose	Safety Measures	Side Effects/Adverse Event (Mostly Reported)	Withdrawal Due to AEs
RUPP., 2005 [35]; Posey et al., 2006 [36]; Scahill et al., 2017 [56]	Placebo	//	survey on side effects filled by parents; physical examination (blood pressure, pulse, body weight, height, temperature)	bradycardia, diarrhea	n. 6 subjects showed intolerable adverse effects with >1 dosage level and dropped out of the test-dose phase. N. 7 exited during the cross-over phases. The highest dose was considered missing for 16 subjects who showed intolerable adverse effects.
	MPH IR	~0.125 mg/kg ×2/day + half dose at 4 P.M.		Sleep difficulties, irritability, emotional outbursts	
		~0.25 mg/kg ×2/day + half dose at 4 P.M.		decreased appetite, sleep difficulties, irritability, and emotional outbursts.	
		~0.5 mg/kg ×2/day + half dose at 4 P.M.		decreased appetite, sleep difficulties, stomach or abdominal discomfort, irritability, and emotional outbursts.	
RUPP., 2005 [35]	MPH IR	adjusted on clinical judgment	survey on side effects filled by parents; physical examination (blood pressure, pulse, body weight, height, temperature)	not specified	n. 3 for adverse effects, lack of efficacy, or declined participation (not specified)
Santosh et al., 2006 [38]	MPH	30 mg/day in 3 divided doses	//	low mood, sleep difficulties, depressive ideas	//
		30 mg/day in 3 divided doses		low mood, sleep difficulties, appetite problems, dysphoria, obsessional behavior	
	MPH	mean dose 31.7 mg/day	NR	nausea, giddiness, headache, sleep difficulties	//
		mean dose 33.8 mg/day		sleep difficulties	
Simonoff et al., 2012 [39]	Placebo	//	Parental interview: sleep difficulties, loss of appetite, sadness, crying spells, anxiety, repetitive behavior, social withdrawal, daydreams, irritable, stomach ache, headache, drowsy, excitability, anger, nightmares, tics, tremors of hands. Medical evaluation: weight, pulse, blood pressure	no significant differences in AE between the EG and CG. The most reported AEs: Sleep difficulties, decreased appetite, weight change	n. 5 participants due to irritability, poor appetite and aggression, nausea and diarrhea, blurred vision, urticaria, and irritability combined with social withdrawal. n. 5 withdrew consent after being randomly assigned to their groups.
	MPH i.r.	0.5 mg/kg ×3/day			
		1.0 mg/kg ×3/day			
		1.5 mg/kg ×3/day			
Pearson et al., 2013; 2020 [40,41]	Placebo	//	Physician desk reference survey on side effects filled by parents	//	//
	MPH (ER morning + IR afternoon doses)	LA 0.21 mg/kg + IR 0.14 mg/kg		//	n. 5 patients discontinued Afternoon IR-MPH for the presence of: irritability (n. 5/5), decreased sleep (n. 2/5), increased stereotyped behaviors (n.2/5)
		0.35 mg/kg + 0.24 mg/kg		//	
		0.48 mg/kg + 0.27 mg/kg		Decreased appetite, sleep difficulties (Pearson D.A. et al., 2013 [40]);	
Golubchik et al., 2017 [42]	MPH	10–54 mg/day	spontaneous self-reports of adverse effects	minor side effects (not specified)	//
Kim et al., 2017 [43]	MPH ER	0.29 mg/kg	HALP Sleep Questionnaire, RISK-K, C-SSRS	Rebound at the end of the day, aggression, and irritability were significantly more reported for medium dose. Other reported sleep difficulties and decreased appetite.	n. 1 withdrawal at week 4 due to time commitment issues
		0.5 mg/kg			
Peled et al., 2019 [44]	MPH IR	10 mg i.r.	//	N.R.	//
Ventura P. et al., 2022 [45]	MPH ER	0.8 mg/kg	//	Decreased appetite, abdominal discomfort, headache, palpitation, irritability, anxiety, insomnia, hyperfocusing	n. 2 ASD patients interrupted treatment for restlessness, increased stereotyped behaviours.
		0.78 mg/kg			
Arnold et al., 2006 [46]	Placebo	//	physical examination, weekly vital signs, weight, EKG, spontaneously reported Aes. Clinician-rated 16-item side effects scale (based on side effects abstracted from the package insert)	no significant differences in AE between the EG and CG. Most frequently reported Heart rate, Upset stomach, Nausea/vomiting, Tiredness/fatigue, Racing heart	//
	ATX	1.4 mg/kg/day in split dose (maximum daily dose)			
Troost et al., 2006 [47]	ATX	0.5–1.8 mg/kg/day (divided into two doses based on patient-preference)	weekly open-ended questioning, vital signs, physical assessment, routine laboratory tests, EKG.	Decreased appetite, irritability, sleep problems, mean heart rate increased	n. 3 gastrointestinal compliance, n. 2 anxiety and increased aggressivity
Posey et al., 2006 [36]	ATX	1.2–1.4 mg/kg/day	open-ended question	sedation, irritability, decreased appetite, decrease/increase thirst	n. 1 swallow pills, n.2 for irritability
Harfeterkamp et al., 2012; 2014 [48,49]	ATX	1.2 mg/kg/day	open-ended questioning	nausea, decreased appetite, sleep difficulties	N. 1 adverse event (fatigue), n. 2 protocol violation, n. 1 no efficacy, n. 1 parents decision
	Placebo	//		//	n. 2 protocol violation, n.1 physician decision
Harfeterkamp et al., 2013 [50]	ATX	0.8–1.2 mg/kg/day, based on tolerability	open-ended questioning	abdominal pain, decreased appetite, fatigue, headache, nausea	n. 11 adverse events, n. 4 lack of efficacy
Fernandez-Jaen et al., 2013 [51]	ATX	39.79 mg/day (±12.46)	//	sleep difficulties, gastrointestinal symptoms, irritability	n. 3 for adverse events and no efficacy, N. 2 for no efficacy.
Handen et al. 2015 [52]; Tumuluru et al., 2017 [53]	ATX + PT	1.35 mg/kg/split twice daily	Side effects checklist (filled by parents), Side effects review (parents and subjects), blood pressure, pulse, height, and weight, EKG, blood work (Tumuluru, E. V., et al., 2017 [53])	The only significant difference between ATX and placebo was the higher rate of decreased appetite in ATX. Most frequently reported: mood swing, restlessness, upset stomach, decreased appetite, constipation, sleep problems	n. 5 patients discontinued due to behavioral difficulties or irritability
	ATX	1.38 mg/kg/split twice daily			
	Placebo + PT	//			n. 10 on placebo withdrew due to increased behavioral difficulties and physical side effects (e.g., GI issues and insomnia).
	Placebo	//			
Smith et al., 2016 [54]	ATX	1.16 mg/kg	this 24 week extension study reported maintenance of responder status in pregresso ATX responders; improvement in adhd symptoms reported in no-placebo responders; no any improvement from acute trial were retrived	decreased appetite, nausea, vomiting, constipation, headache, mood swing, sleep difficulties, fatigue	n. 4 behavioral deterioration, n. 8 side-effects, n. 2 parent request
	Placebo	//		//	
	ATX + PT	1.16 mg/kg		decreased appetite, nausea, vomiting, constipation, headache, mood swing, sleep difficulties, fatigue	
	Placebo + PT	//		//	
Kilincaslan et al. 2016 [55]	ATX	1.20 ± 0.11 mg/kg/day	BSERS	uninterested in others, drowsiness, dizziness, increased blood pressure	n.5 adverse events (increased motor activity, talkativeness, irritability, temper outbursts, increased blood pressure)
Scahill et al., 2006 [56]; McCracken et al., 2010 [57]	Guanfacine	3–5 mg/day	vital signs, height and weight, systematic review of adverse events (Scahill, L et al. 2006 [56])	Sedation, Irritability, sleep difficulties	NR
Scalhill et al., 2015 [58]; Politte, et al. 2018 [59]	ER Guanfacine	3–4 mg/day	Blood pressure, pulse, height, weight, 34-item question on AE	Drowsiness, fatigue, decreased appetite, dry mouth, emotional/tearful, irritability, anxiety, sleep difficulties	N.2 subjects due to AEs, N.2 due to lack efficacy
	Placebo	//		headache, excessive talking, increased energy	N. 4 subjects due to lack of efficacy
Jaselskis et al., 1992 [60]	Placebo	//	//	//	//
	Clonidine	0.15–0.20 mg/day	blood pressure, symptom checklist	hypotension, irritability, drowsiness, decreased activity, sleep difficulties	
Lamberti et al., 2016 [61]	Risperidone	3 mg/day	ECG, blood pressure, pulse, body weight, height, body mass index, abdominal circumference, laboratory test (fasting blood glucose, insulin and lipid levels, prolactin, other general blood tests). AIMS	n.11 increased appetite, n.8 weight gain, n.4 drowsiness; n.3 higher plasma prolactin level	n.1 lack of compliance, n.1 decline to return, n.1 restlessness with akatisia
	Aripiprazole	15 mg/day		n.5 increased appetite, n.4 weight gain, n.4 drowsiness	n.1 lack of compliance, n. 2 decline to return, n. 1 restlessness with sleep difficulties

Pharmacological agent/group legend: MPH = Methylphenidate; ATX = atomoxetine; IR = Immediate Release; ER = Extended Release; PT = Parent Training; EKG = Electriocardiogram; EG = Experimental Group; CG = Control Group; RISC-K = Response Impressions and Side Effects Check-list-Kids; C-SSRS = Columbia Suicide Severity Rating Scale; HALP = hyperactivity, attention, learning problems; BSERS = Barkley Side Effect Rating Scale; N.R. = No Reported.

**Table 4:** Characteristics of non-pharmacological interventions reported in included studies.

Reference	Study Design	Sample Size	Ethnicity %	Sex Ratio M:F	Age Range in Years (Mean ± SD)	Mean IQ (SD)	Investigated Domains	Outcome Measures	Treatment	N. Sessions (Duration; Frequency)	Therapeutic Effects
Chan et al., 2023 [66]	RCT	95 (26 ASD; 24 ASD + ADHD; 22 ASD control; 23 non-autistic)	100% Chinese	ASD 12:1 ASD + ADHD 24:0 ASD: 4.5:1 Non autism 10.5:1	4–6 (5.11 ± 0.87)	100–105 (11.71)	Emotion recognition	EVT; ERT1/2/3	The Transporters—App-based emotion recognition training: daily viewing of animated episodes (15 emotions) using vehicle characters with human faces in social story contexts	28 days (15–20 min/day; daily for 4 weeks)	Significant improvement in emotion recognition in both ASD and ASD + ADHD groups vs. control; Learning was generalizable to novel situations.
Chu et al., 2023 [67]	RCT	78 (53 ASD+ ADHD)	100% Chinese	3.6:1	3–6 (5.02 ± 0.52)	IQ > 70	Autism symptoms, inattention, hyperactivity, impulsivity, response inhibition	ABC, CARS, ADHD-RS-IV, Go/No-Go task (accuracy and reaction time)	VR-CBT: Virtual reality-based cognitive-behavioral therapy with interactive games projected on the floor to train attention, inhibition and social skills; LSP: Structured therapist-led program that adapts learning strategies to individual profiles.	40 sessions (2×/week; 20 min VR-CBT + 1 h LSP per session for 20 weeks)	In the ASD + ADHD subgroup, significant reduction in hyperactivity-impulsivity severity and in total scores of ADHD-RS-IV. No significant change in inattention symptoms.
Patel et al., 2007 [68]	open-label observational	10	NR	9:1	4–10 (5.9)	NR	motor, social, behavioral, and educational skills, symptoms of autism and ADHD, Urinary metals, and metabolic analysis	motor, behavioral, and educational skills evaluated through an ad hoc questionnaire rated by parents, teachers, and the physician; blood laboratory tests, urinary heavy metals, intradermal testing, organic acid analysis	Multidimensional treatment protocol: (I) environmental control and avoidance of triggers; (II) an organic diet; (III) gastrointestinal support; (IV) antigen injection therapy; (V) nutritional supplements; (VI) chelation therapy; (VI) glutathione and methylcobalamin integration; (VII) usual therapies.	3–6 months	Parents reported improvement in social interaction, concentration, writing, language, and behaviors. Urinary lead burden decreased significantly
Yerys et al., 2018 [69]	RCT	11	9.1% Black 9.1% Multir.72.7% White 9.1% NR	11:0	9–13 (11.25 ± 1.12)	98.36 (13.11)	sustained attention, inattention, hyperactivity, impulsivity, Behavior Regulation, Emotion Regulation, Cognitive Regulation, psychomotor speed and accuracy, retaining and updating visuospatial information, social skills, problem behaviors	TOVA, CANTAB, ADHD Rating Scale-IV, BRIEF-2, SSIS.	Project Evo—multi-tasking gameplay: a perceptual discrimination attention/memory task and a continuous visuomotor driving-type task on a tablet.	20 sessions (25 min; five times a week for 4 weeks)	Significant improvement in ADHD-RS-index, Behavior Regulation, Emotion Regulation, Cognitive Regulation, social skills, and Problem Behaviors.
		8	12.5% Multir.87.5% White	6:2	9–13 (11.26 ± 1.88)	111.12 (16.99)			educational-based intervention: a task that requires children to generate words from an array of letters on a tablet		//

TOVA = Test of Variables of Attention; CANTAB = Cambridge Neuropsychological Test Automated Battery; SSIS = Social Skills Improvement System; ADHD Rating Scale-IV, BRIEF-2 = Behavior Regulation Inventory of Executive Function; N.R. = No Reported; EVT = Emotion Vocabulary Task; ERT1/2/3 = Emotion Recognition Tasks—levels of generalization; ABC = Autism Behavior Checklist; Cars = Childhood Autism Rating Scale.

**Table 5:** Quality assessment of included studies.

Publication	Exposed Representation	Ascertainment of Exposure	Selection of the Non-Exposed	Outcome Was Not Present at Start of Study	Comparability of Cohorts	Assessment of Outcome	Sufficient Follow-Up Time	Adequacy of Follow-Up of Cohorts	Total
Posey et al., 2006 [36] **(A)**	0	1	0	0	0	1	0	1	3
Santosh et al., 2006 [38] **(M)**	1	1	1	0	0	1	0	1	5
Scahill et al., 2006 [56] **(G)**	1	0	1	1	0	0	0	0	3
Troost et al., 2006 [47] **(A)**	1	1	1	0	0	1	0	0	4
Patel et al., 2007 [68] **(NP)**	1	1	0	0	0	1	1	0	4
McCracken et al., 2010 [57] **(G)**	1	1	0	0	0	1	1	1	5
Fernández-Jaén et al., 2013 [51] **(A)**	1	1	0	0	0	1	1	1	5
Kilincaslan et al., 2016 [55] **(A)**	1	1	0	0	0	1	0	1	4
Lamberti et al.,2016 [61] **(R)**	1	1	0	0	0	1	1	1	5
Golubchik et al.,2017 [42] **(M)**	1	1	1	0	0	0	0	0	3
Yerys et al., 2018 [69] **(NP)**	1	1	0	0	0	1	0	0	3
Peled et al., 2019 [44] **(M)**	0	1	0	1	0	1	0	1	4
Ventura et al., 2022 [45] **(M)**	1	0	0	0	0	1	1	0	3

A = Atomoxetine; M = Methylphenidate; G = Guanfacine, R = Risperidone; NP = non-pharmacological.

**Table 6:** Risk domains for each included study.

Publication	Random Sequence Generation	Allocation Concealment	Selective Reporting	Other Bias	Blinding of Participants and Personnel	Blinding of Outcome Assessment	Incomplete Outcome Data	AHRQ Standard
Jaselskis et al.,1992 [60] **(C)**	Low	Low	Unclear	Unclear	Low	Low	Unclear	Poor
Arnold et al., 2006 [46] **(A)**	High	High	Unclear	Unclear	Low	Unclear	Low	Poor
Handen et al., 2006 [52] **(A)**	Low	Low	Unclear	Unclear	Low	Low	Unclear	Fair
Posey et al., 2007 [36] **(M)**	High	High	Unclear	Unclear	Low	Low	Low	Poor
Harfterkamp et al., 2012 [48] **(A)**	Low	Low	Low	Unclear	Low	Low	Unclear	Fair
Simonoff et al.,2012 [39] **(M)**	Low	Low	Low	Low	Low	Low	Low	Good
Harfterkamp et al., 2013 [50] **(A)**	Unclear	Unclear	Unclear	Unclear	Low	Unclear	Low	Poor
Pearson et al., 2013 [40] **(M)**	Unclear	Low	Unclear	Low	Low	Low	Low	Fair
Harfterkamp et al., 2014 [49] **(A)**	Low	Low	Low	Unclear	Low	Unclear	Low	Fair
Scahill et al., 2015 [58] **(G)**	Low	Low	Low	Low	Low	Low	Low	Good
Kim et al., 2017 [43] **(M)**	Unclear	Low	Unclear	Unclear	Low	Unclear	Low	Poor
Scahill et al., 2017 [37] **(M)**	Unclear	Unclear	Unclear	Unclear	Low	Low	Low	Poor
Smith et al., 2017 [54] **(A)**	Unclear	Unclear	Unclear	Unclear	Low	Unclear	Low	Poor
Tumuluru et al., 2017 [53] **(A)**	High	High	Unclear	Unclear	Low	Unclear	Unclear	Poor
Politte et al., 2018 [59] **(G)**	Low	Unclear	Low	Unclear	Low	Unclear	Unclear	Poor
Pearson et al., 2020 [41] **(M)**	Unclear	Low	Unclear	Unclear	Low	Low	Low	Poor
Chu et al.,2023 [67] **(NP)**	Low	Low	Unclear	Unclear	High	High	Low	Poor
Chan et al.2024 [66] **(NP)**	Unclear	Low	Unclear	Unclear	High	High	Low	Poor

A = Atomoxetine; C = Clonidine; M = Methylphenidate; G = Guanfacine, R = Risperidone; NP = non-pharmacological.

## Data Availability

The data presented in this study are available on request from the corresponding author.

## Supplementary Materials

The following supporting information can be downloaded at: https://www.mdpi.com/article/10.3390/jcm14114000/s1, Supplement S1: References for included studies; Table S1: PRISMA 2020 statement and checklist; Supplement S2: Literature search. This supplementary material has been provided by the authors to give readers additional information about their work.

## Abbreviations

The following abbreviations are used in this manuscript:

ADHD	Attention-deficit/hyperactivity Disorder
ASD	Autism Spectrum Disorder
MPH	Methylphenidate
ATX	Atomoxetine
GFC	Guanfacina
DSM	Diagnostic and Statistical Manual of Mental Disorders
RCTs	Randomized Controlled Studies
ICD	International Classification of Diseases
IQ	Intellectual Quotient
PRISMA	Preferred Reporting Items for Systematic reviews and Meta-Analyses
NOS	Newcastle-Ottawa Scale
AHRQ	Agency for Healthcare Research and Quality
ABC	Aberrant Behavior Checklist
CPRS	Conners Parent Rating Scales–Revised
SNAPIV	Swanson, Noland, and Pelham Scale IV
CGI	Clinical Global Improvement
AE	Adverse Events
VR-CBT	Virtual reality-based cognitive-behavioral therapy
LSP	Therapist-led program
PT	Parent Training
RIS	Risperidone
ARI	Aripiprazole
CONSORT	Consolidated Standards of Reporting Trials
ED	Emotion Dysregulation
