# Supplementary material for: Children and Adolescents with Co-Occurring Attention-Deficit/Hyperactivity Disorder and Autism Spectrum Disorder: A Systematic Review of Multimodal Interventions
Source: J Clin Med. 2025 Jun 5;14(11):4000. doi: 10.3390/jcm14114000 (PMC12156790; doi:10.3390/jcm14114000)
Supplement: Supplementary file 1 [file jcm-14-04000-s001.zip › jcm-3622403-supplementary.pdf]

SUPPLEMENTARY MATERIAL

Supplement 1: References for included studies……..page 2-6

Table S1: PRISMA 2020 statement and checklist………..page 7-9

Supplement 2: Literature search………page 10

This supplementary material has been provided by the authors to give readers additional information about their work.

Supplement 1

Author	Year	Title	Journal	Country
Arnold et al.	2006	Atomoxetine for Hyperactivity in Autism Spectrum Disorders: Placebo-Controlled Crossover Pilot Trial	*American Academy Of Child And Adolescentpsychiatry*	*North America, Ohio*
Chan et al.	2024	Enhancing emotion recognition in young autistic children with or without attention-deficit/hyperactivity disorder in Hong Kong using a Chinese App version of The Transporters	*Autism*	*Asia, Hong Kong*
Chu et al.	2023	Effects of a Nonwearable Digital Therapeutic Intervention on Preschoolers With Autism Spectrum Disorder in China: Open-Label Randomized Controlled Trial	*Journal of Medical Internet Research*	*Asia, China*
Fernandez-Jaen et al.	2013	Efficacy of Atomoxetine for the Treatment of ADHD Symptoms in Patients With Pervasive Developmental Disorders: A Prospective, Open-Label Study	*Journal Of Attention Disorders*	*Europe, Spain*
Golubchik et al.	2017	The effect of methylphenidate on anxiety and depression symptoms in patients with Asperger syndrome and comorbid attention deficit/hyperactivity disorder	*International Clinical Psychopharmacology*	*Asia, Israel*
Handen et al.	2015	Atomoxetine, Parent Training, and Their Combination in Children With Autism Spectrum Disorder and Attention-Deficit/ Hyperactivity Disorder	*American Academy Of Child And Adolescent psychiatry*	*North America,* Pennsylvania; *Ohio; New York*
Harfeterkamp et al.	2012	A Randomized Double-Blind Study of Atomoxetine Versus Placebo for AttentionDeficit/Hyperactivity Disorder Symptoms in Children With Autism Spectrum Disorder	*Journal Of The American Academy Of Child & Adolescent Psychiatry*	*Europe, Netherlands*
Harfeterkamp et al.	2013	Long-Term Treatment with Atomoxetine for Attention-Deficit/Hyperactivity Disorder Symptoms in Children and Adolescents with Autism Spectrum Disorder: An Open-Label Extension Study	*Journal Of Child And Adolescent Psychopharmacology*	*Europe, Netherlands*

Harfeterkamp et al.	2014	Atomoxetine in Autism Spectrum Disorder: No Effects on Social Functioning; Some Beneficial Effects on Stereotyped Behaviors, Inappropriate Speech, and Fear of Change	*Journal Of Child And Adolescent Psychopharmacology*	*Europe, Netherlands*
Jaselskis et al.	1992	*Clonidine Treatment of hyperactive and Impulsive Children With Autistic Disorder*	*Journal Of Clinical Psychopharmacology*	*North America, Illinois*
Kilincaslan et al.	2016	Effects of Atomoxetine in Individuals with Attention-Deficit/Hyperactivity Disorder and Low-Functioning Autism Spectrum Disorder	*Journal Of Child And Adolescent Psychopharmacology*	*Europe, Turkey*
Kim et al.	2017	Dose-Response Effects of Long-Acting Liquid Methylphenidate in Children with Attention Deficit/Hyperactivity Disorder (ADHD) and Autism Spectrum Disorder (ASD): A Pilot Study	*Journal of Autism and Developmental Disorders*	*North America, Washington*
Lamberti M.	2016	Head-to-Head Comparison of Aripiprazole and Risperidone in the Treatment of ADHD Symptoms in Children with Autistic Spectrum Disorder and ADHD: A Pilot, Open-Label, Randomized Controlled Study	*Pediatric Drugs*	*Europe, Italy*
McCracken et al.	2010	PossibleInfluence of Variant of the P-Glycoprotein Gene (MDR1/ABCB1) on ClinicalResponse to Guanfacine in Children with Pervasive Developmental Disorders and Hyperactivity	*Journal Of Child And Adolescent Psychopharmacology*	*North America, California*
Patel K. et al.	2007	A Comprehensive Approach to TreatingAutism and Attention-Deficit Hyperactivity Disorder: A Prepilot Study	*The Journal Of Alternative And Complementary Medicine*	*North America, NewYork*

Pearson et al.	2020	Attentional shift within and between faces: Evidence from children with and without a diagnosis of autism spectrum disorder	*Journal Of Child And Adolescent Psychopharmacology*	*North America, Texas*
Pearson et al., 2013	2013	Effects of Extended Release Methylphenidate Treatment on Ratings of Attention-Deficit/Hyperactivity Disorder (ADHD) and Associated Behavior in Children with Autism Spectrum Disorders and ADHD Symptoms	*Journal Of Child And Adolescent Psychopharmacology*	*North America, Texas*
Peled et al.	2019	Processing speed as a marker to stimulant effect in clinical sample of children with high functioningautism spectrum disorder	*Nordic Journal Of Psychiatry*	*Asia, Israel*
Politte et al.	2018	A randomized, placebo-controlled trial of extended-release guanfacine in children with autism spectrum disorder and ADHD symptoms: an analysis of secondaryoutcome measures	*Neuropsychopharmacology*	*North America, California; Georgia; Massachusetts; Washington; Connecticut*
Posey et al.	2006	Open-Label Atomoxetine for Attention-Deficit/ Hyperactivity Disorder Symptoms Associated with High-Functioning Pervasive Developmental Disorders	*Journal Of Child And Adolescent Psychopharmacology*	*North America, Indiana*
Posey et al.	2007	Positive Effects of Methylphenidate on Inattention and Hyperactivity in Pervasive Developmental Disorders: An Analysis of Secondary Measures	*Biological Psychiatry*	*North America, Indiana; Maryland; Ohio; California; Connecticut*
RUPP.	2005	Randomized, Controlled, Crossover Trial of Methylphenidate in Pervasive Developmental Disorders With Hyperactivity	*Archives of General Psychiatry*	*North America, Indiana; Maryland; Ohio; California; Connecticut*

Santosh et al.	2006	Impact of comorbidautism spectrum disorders on stimulantresponse in childrenwith attention deficit hyperactivity disorder: a retrospective and prospectiveeffectiveness study	*Journal Compilation*	*Europe, England*
Scahill et al.	2006	A Prospective Open Trial of Guanfacine in Children with Pervasive Developmental Disorders	*Journal Of Child And Adolescent Psychopharmacology*	*North America, Ohio*
Scahill et al.	2017	Using a Patient-CenteredOutcome Measure to Test Methylphenidate Versus Placebo in Children with Autism Spectrum Disorder	*Journal Of Child And Adolescent Psychopharmacology*	*North America, Indiana; Maryland; Ohio; California; Connecticut*
Scailhill et al.	2015	Extended-Release Guanfacine for Hyperactivity in Children With Autism Spectrum Disorder	*American Journal of Psychiatry in Advance*	*North America, California; Georgia; Massachusetts; Washington; Connecticut*
Simonoff et al.,	2012	Randomizedcontrolled double-blind trial of optimal dose methylphenidate in children and adolescents with severe attention deficit hyperactivity disorder and intellectual disability	*Journal Of Child Psychology And Psychiatry*	*Europe, England*
Smith et al.	2016	Atomoxetine and Parent Training for Children With Autism and Attention-Deficit/Hyperactivity Disorder: A 24-Week Extension Study	*American Academy Of Child And Adolescent psychiatry*	*North America,* Pennsylvania; *Ohio; New York*
Troost et al.	2006	Atomoxetine for Attention-Deficit/Hyperactivity Disorder Symptoms in Children with Pervasive Developmental Disorders: A Pilot Study	*Journal Of Child And Adolescent Psychopharmacology*	*Europe, Netherlands*

Tumuluru et al.	2017	AdverseEvents of Atomoxetine in a Double-Blind Placebo-Controlled Study in Children with Autism	*American Academy Of Child And Adolescentpsychiatry*	*North America,* Pennsylvania; *Ohio; New York*
Ventura et al.	2022	Methylphenidate Use for Emotional Dysregulation in Children and Adolescents with ADHD and ADHD and ASD: A Naturalistic Study	*Journal Of Clinical Medicine*	*Europe, Italy*
Yerys et al.	2018	Brief Report: Pilot Study of a Novel Interactive Digital Treatment to Improve Cognitive Control in Children with Autism Spectrum Disorder and Co-occurring ADHD Symptoms	*Journal Of Autism And Developmental Disorders*	*North America, Pennsylvania*

Table S1: PRISMA 2020 statement and checklist

Section and topic	Item #	Checklist item	Location where item is reported
**TITLE**			
Title	1	Identify the report as a systematic review.	Title
**ABSTRACT**			
Structured summary	2	See the PRISMA 2020 for Abstracts checklist.	Abstract
**INTRODUCTION**			
Rationale	3	Describe the rationale for the review in the context of existing knowledge.	Introduction
Objectives	4	Provide an explicit statement of the objective(s) or question(s) the review addresses.	Introduction
**METHODS**			
Eligibility criteria	5	Specify the inclusion and exclusion criteria for the review and how studies were grouped for the syntheses.	Methods
Information sources	6	Specify all databases, registers, websites, organisations, reference lists and other sources searched or consulted to identify studies. Specify the date when each source was last searched or consulted.	Methods
Search strategy	7	Present the full search strategies for all databases, registers and websites, including any filters and limits used.	Methods, Supplementary 1
Selection process	8	Specify the methods used to decide whether a study met the inclusion criteria of the review, including how many reviewers screened each record and each report retrieved, whether they worked independently, and if applicable, details of automation tools used in the process.	Methods
Data collection process	9	Specify the methods used to collect data from reports, including how many reviewers collected data from each report, whether they worked independently, any processes for obtaining or confirming data from study investigators, and if applicable, details of automation tools used in the process.	Methods, Figure 1
Data items	10a	List and define all outcomes for which data were sought. Specify whether all results that were compatible with each outcome domain in each study were sought (e.g. for all measures, time points, analyses), and if not, the methods used to decide which results to collect.	Methods
	10b	List and define all other variables for which data were sought (e.g. participant and intervention characteristics, funding source). Describe any assumptions made about any missing or unclear information.	Methods

Study risk of bias assessment	11	Specify the methods used to assess risk of bias in the included studies, including details of the tool(s) used, how many reviewers assessed each study and whether they worked independently, and if applicable, details of automation tools used in the process.	Methods
Effect measures	12	Specify for each outcome the effect measure(s) (e.g. risk ratio, mean difference) used in the synthesis or presentation of results.	Methods
Synthesis methods	13a	Describe the processes used to decide which studies were eligible for each synthesis (e.g. tabulating the study intervention characteristics and comparing against the planned groups for each synthesis (item #5)).	Methods
	13b	Describe any methods required to prepare the data for presentation or synthesis, such as handling of missing summary statistics, or data conversions.	Does not apply
	13c	Describe any methods used to tabulate or visually display results of individual studies and syntheses.	Methods
	13d	Describe any methods used to synthesise results and provide a rationale for the choice(s). If meta-analysis was performed, describe the model(s), method(s) to identify the presence and extent of statistical heterogeneity, and software package(s) used.	Methods
	13e	Describe any methods used to explore possible causes of heterogeneity among study results (e.g. subgroup analysis, metaregression).	Methods
	13f	Describe any sensitivity analyses conducted to assess robustness of the synthesised results. Reporting bias assessment	Methods
Reporting bias	14	Describe any methods used to assess risk of bias due to missing results in a synthesis (arising from reporting biases).	Methods
Certainty assessment	15	Describe any methods used to assess certainty (or confidence) in the body of evidence for an outcome	Does not apply
**RESULTS**			
Study selection	16a	Describe the results of the search and selection process, from the number of records identified in the search to the number of studies included in the review, ideally using a flow diagram (see fig 1).	Results, Figure 1 (PRISMA)
	16b	Cite studies that might appear to meet the inclusion criteria, but which were excluded, and explain why they were excluded.	Methods, FIGURE 1 (PRISMA)
Study characteristics	17	Cite each included study and present its characteristics.	Results
Risk of bias within studies	18	Present assessments of risk of bias for each included study	Results, Table 6
Results of individual studies	19	For all outcomes, present, for each study: (a) summary statistics for each group (where appropriate) and (b) an effect estimate and its precision (e.g. confidence/credible interval), ideally using structured tables or plots.	Does not apply
Results of syntheses	20a	For each synthesis, briefly summarise the characteristics and risk of bias among contributing studies.	Results, Table 2, Table 6

	20b	Present results of all statistical syntheses conducted. If meta-analysis was done, present for each the summary estimate and its precision (e.g. confidence/credible interval) and measures of statistical heterogeneity. If comparing groups, describe the direction of the effect.	Does not apply
	20c	Present results of all investigations of possible causes of heterogeneity among study results.	Results, Table 2,
	20d	Present results of all sensitivity analyses conducted to assess the robustness of the synthesised results	Results, Table 2
Reporting biases	21	Present assessments of risk of bias due to missing results (arising from reporting biases) for each synthesis assessed.	Results, Table 6
Certainty of evidence	22	Present assessments of certainty (or confidence) in the body of evidence for each outcome assessed.	Results, Table 4
**DISCUSSION**			
Discussion	23a	Provide a general interpretation of the results in the context of other evidence.	Discussion
	23b	Discuss any limitations of the evidence included in the review.	Discussion
	23c	Discuss any limitations of the review processes used.	Discussion
	23d	Discuss implications of the results for practice, policy, and future research.	Discussion
**OTHER INFORMATION**			
Registration and protocol	24a	Provide registration information for the review, including register name and registration number, or state that the review was not registered.	Methods
	24b	Indicate where the review protocol can be accessed, or state that a protocol was not prepared.	Methods
	24c	Describe and explain any amendments to information provided at registration or in the protocol.	Does not apply
Support	25	Describe sources of financial or non-financial support for the review, and the role of the funders or sponsors in the review.	Funding
Competing interests	26	Declare any competing interests of review authors.	Conflict of interest
Availability of data, code, and other	27	Report which of the following are publicly available and where they can be found: template data collection forms; data extracted from included studies; data used for all analyses; analytic code; any other materials used in the review.	Does not apply

Supplement 2: Literature search

The following search terms were applied:

autism: "autism's" OR "autisms" OR "autistic spectrum disorder" OR "autistic disorder" OR "ASD" OR "pervasive development disorder" OR "Asperger" OR "Asperger syndrome" OR "childhood disintegrative disorder".

adhd: "attention deficit disorder with hyperactivity" OR "adhd" OR "attention deficit" OR "attention disorder" OR "hyperactivity" OR "attention deficit disorder with hyperactivity".

treatment: "therapeutics" OR "intervention" OR "treatment" OR "therapy" OR "treatment's" OR "pharmacological intervention" OR "rehabilitation" OR "non pharmacological" OR "pharmacological therapy" OR "behavioral intervention"
